# Integrin-linked kinase regulates the niche of quiescent epidermal stem cells

**DOI:** 10.1038/ncomms9198

**Published:** 2015-09-08

**Authors:** Jessica Morgner, Sushmita Ghatak, Tobias Jakobi, Christoph Dieterich, Monique Aumailley, Sara A. Wickström

**Affiliations:** 1 Paul Gerson Unna Group ‘Skin Homeostasis and Ageing', Max Planck Institute for Biology of Ageing, Cologne 50931, Germany; 2 Computational RNA Biology and Ageing, Max Planck Institute for Biology of Ageing, Cologne 50931, Germany; 3 Center for Biochemistry, Medical Faculty, University of Cologne, Cologne 50931, Germany; 4 Cologne Excellence Cluster on Cellular Stress Responses in Aging-Associated Diseases (CECAD), University of Cologne, Cologne 50931, Germany

## Abstract

Stem cells reside in specialized niches that are critical for their function. Quiescent hair follicle stem cells (HFSCs) are confined within the bulge niche, but how the molecular composition of the niche regulates stem cell behaviour is poorly understood. Here we show that integrin-linked kinase (ILK) is a key regulator of the bulge extracellular matrix microenvironment, thereby governing the activation and maintenance of HFSCs. ILK mediates deposition of inverse laminin (LN)-332 and LN-511 gradients within the basement membrane (BM) wrapping the hair follicles. The precise BM composition tunes activities of Wnt and transforming growth factor-β pathways and subsequently regulates HFSC activation. Notably, reconstituting an optimal LN microenvironment restores the altered signalling in ILK-deficient cells. Aberrant stem cell activation in ILK-deficient epidermis leads to increased replicative stress, predisposing the tissue to carcinogenesis. Overall, our findings uncover a critical role for the BM niche in regulating stem cell activation and thereby skin homeostasis.

## Abstract (web summary)

Integrin-linked kinase (ILK) is known to modulate the extracellular matrix and hair follicle morphogenesis. Here, Morgner *et al.* show that lack of ILK causes an aberrant ratio of basement membrane laminins, activating stem cells and predisposing skin to carcinogenesis.

## Graphical abstract

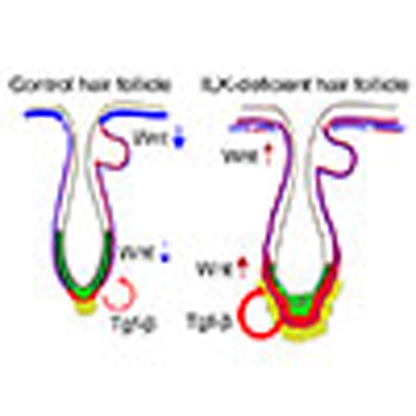

Somatic stem cells (SCs) play key roles in replenishing tissues with differentiated cells that are lost during homeostatic renewal or during injury. SCs are established and maintained in specific anatomic locations termed niches, which are essential for SC homeostasis. Niches integrate signals that mediate the balanced response of SCs to the needs of organisms, prevent SC depletion and at the same time restrict excessive SC expansion into the surrounding tissue^1^.

The epidermis is an ideal tissue to explore the dynamic interactions between SCs and their niche. Mouse epidermis is composed of a pilosebaceous unit that consists of the hair follicle (HF), the sebaceous gland and the surrounding interfollicular epidermis (IFE). Each unit contains multiple distinct SC populations that fuel the constant renewal of the IFE and HFs during postnatal tissue homeostasis and regeneration^2^. The bulge region of the HF harbours quiescent HFSCs that undergo periodical activation to induce a so-called hair cycle, which commences with a growth (anagen) phase, followed by an involution (catagen) phase and terminated by a resting (telogen) phase. When a subset of HFSCs is activated at the onset of anagen, they leave the niche to generate the hair germ (HG) that grows downwards into the dermis^3^.

Tight regulation of quiescence and activation is required for maintaining a stable pool of HFSCs^4^. The core signalling pathways that regulate SC self-renewal, quiescence and activation have been identified. The bone morphogenetic protein (Bmp) pathway is required for maintaining HFSC quiescence, whereas Wnt and transforming growth factor-β2 (Tgf-β2) pathways are central for SC activation and differentiation^2^. However, it is not clear how these signals are initiated, amplified and switched off to achieve precise spatiotemporal coordination of SC activation and quiescence.

The extracellular matrix (ECM), in particular the specialized ECM forming the basement membrane (BM) of the dermal–epidermal junction, is a major constituent of the HFSC niche^5^. It provides biomechanical cues to the SCs but also regulates signalling by activating diverse cell surface receptors and by acting as a reservoir for growth factors, thereby modulating their bioavailability and activation^6^. The epidermal BM is a dynamic structure that is generated by both keratinocytes and dermal mesenchymal cells. In addition to synthesis, both cell types constantly remodel and organize BM components into a precise configuration^7^,^8^,^9^. Cell–matrix adhesion molecules, including integrins and their cytoplasmic effectors, are central players in this process^10^. Coupling of integrin adhesions to the cytoskeleton is central for precise spatiotemporal control of ECM deposition and remodelling. Integrins bind actin indirectly through the recruitment of a large number of actin-binding and regulatory proteins, such as integrin-linked kinase (ILK). ILK is one of the central regulators of the integrin–actin linkage^11^, and deletion of ILK in mice results in peri-implantation lethality due to severe defects in F-actin organization^12^. On the cellular level, ILK deficiency leads to compromised cell spreading and migration due to defective recruitment of cytoskeletal proteins to adhesion sites, loss of cellular force generation and defects in ECM deposition^12^,^13^,^14^. Mice lacking ILK in the epidermis display epidermal hyperthickening that is associated with accumulation of undifferentiated epidermal progenitor cells (EPCs) and increased proliferation^15^,^16^. In contrast, the role of ILK in HFSCs has not been addressed.

In the present study, we use the HF as a paradigm to show that ILK is required for maintaining the SC niche. Deletion of ILK leads to enhanced activation of HFSCs, ultimately resulting in their exhaustion. This is due to altered remodelling of the BM within the SC niche, promoting sustained activation of Wnt and Tgf-β2 pathways and a failure to re-establish quiescence. The enhanced SC activity leads to increased replicative stress and DNA damage, which predisposes the ILK-deficient epidermis to skin carcinogenesis. Taken together, our data indicate that ILK-mediated remodelling of the niche plays an essential role in SC fate regulation and skin homeostasis.

## Results

### Deletion of ILK leads to progressive loss of quiescent bulge SCs

**Figure 1:**
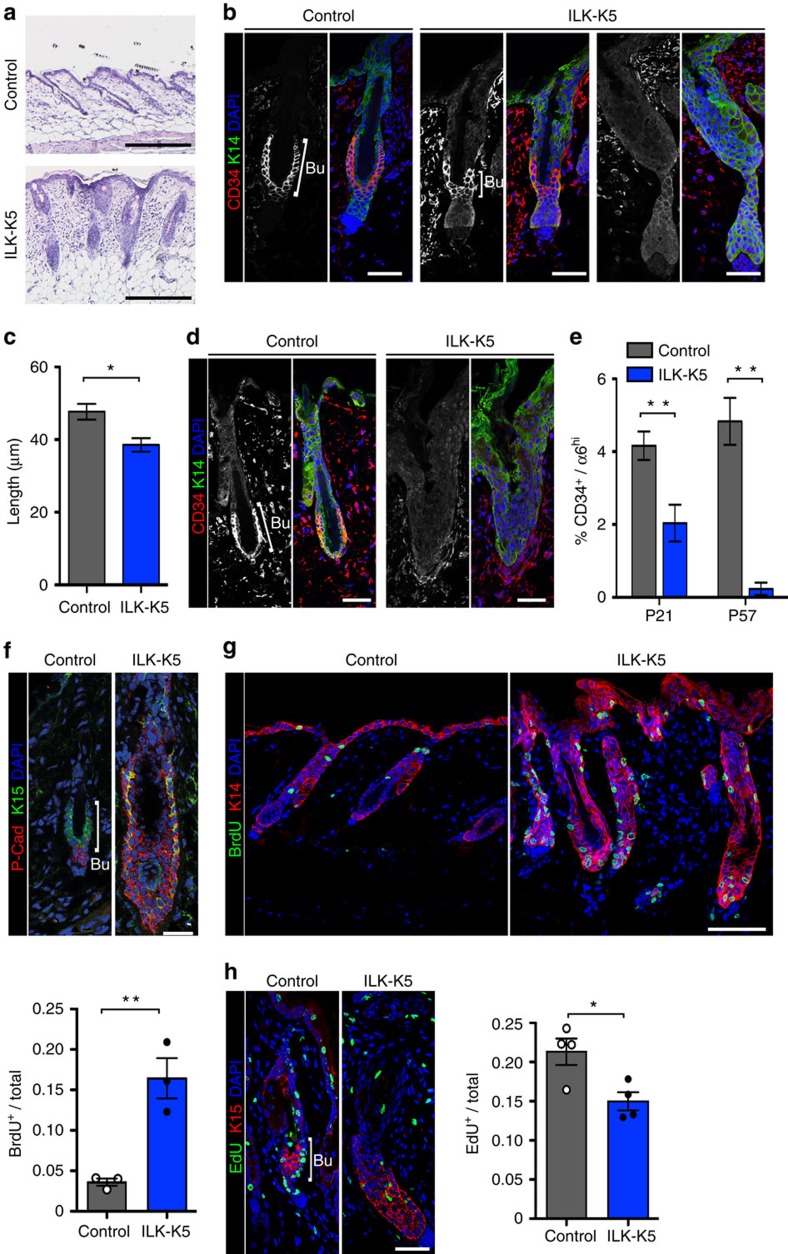
Deletion of ILK leads to progressive loss of quiescent bulge SCs. (**a**) Haematoxylin/eosin staining of P21 skin. Compared with control HFs that display typical telogen morphology, ILK-K5 HFs are thick and more elongated, extending to the subcutis. Scale bar, 200 μm. (**b**) Immunofluorescence staining for the bulge SC marker CD34 (red) and EPC marker K14 (green) from P21 skin. CD34 staining within the bulge (Bu) is decreased or absent (right panel) in ILK-K5 HFs. Scale bars, 30 μm. (**c**) Quantification of bulge length from CD34 stainings at P21. Only HFs where CD34 staining was clearly present were measured (mean±s.e.m.; *n*=3; *P=0.05, Mann–Whitney). (**d**) CD34 staining from P57 skin shows absence of CD34-positive bulge SCs in ILK-K5 HFs. Scale bars, 30 μm. (**e**) FACS analysis of CD34^+^/α6 integrin^hi^ bulge SCs shows progressive reduction of these cells in ILK-K5 skin (mean±s.e.m.; *n*=8; **P=0.0042 for P21; *n*=5; **P=0.0079 for P57; Mann–Whitney). (**f**) Immunofluorescence analysis of the HF progenitor marker K15 (green) and the TAC marker P-Cadherin (P-Cad; red) in P21 HFs shows expansion of both markers and mixing of the K15 and P-cad-positive compartments. Scale bars, 30 μm. (**g**) Detection of BrdU-positive cells within HFs of P21 mice after a 1 h BrdU pulse shows increased levels of BrdU-positive cells in ILK-K5 HFs. Scale bars, 50 μm. Values in quantification represent mean±s.e.m. of BrdU-positive cells per total HF cells (*n*=3; **P=0.0036, Mann–Whitney). (**h**) Analysis of EdU-positive LRCs within HFs of P21 mice after 10 days of EdU chase. Immunostaining shows decreased presence of LRCs in ILK-K5 bulge (Bu) SCs. Scale bars, 30 μm. Values in quantification represent mean±s.e.m. of EdU-positive cells per total cells in HFs (*n*=4; *P=0.05, Mann–Whitney). DAPI, 4,6-diamidino-2-phenylindole.

The most prominent phenotype of mice lacking ILK in their epidermis is progressive hair loss, which occurs after the first postnatal hair cycle at the age of 6–8 weeks (wk)^15^, but the underlying mechanism is not known. As hair cycling is fuelled by HFSCs, we sought to investigate whether ILK regulates postnatal HFSC fate. We analysed HF morphology in ILK^fl/fl^ mice intercrossed with mice expressing Cre under the keratin (K)-5 promoter (from here on termed ILK-K5) during the first telogen phase after morphogenesis (postnatal day (P)21). At this stage, control HFs displayed typical resting-phase architecture characterized by thin, short HFs that terminated directly below the bulge (Fig. 1a). In contrast, most ILK-deficient HFs were thick and elongated, extending to the subcutis (Fig. 1a). Immunofluorescence analysis confirmed efficient deletion of ILK at this point (Supplementary Fig. 1a).

To analyse the HFSC compartment, we stained sections from P21 mice with antibodies against the bulge SC marker CD34 and observed a reduction in CD34 staining in ILK-K5 HFs (Fig. 1b,c). This reduction was progressive and at P57, when most HFs were lost, the remaining HFs showed no CD34 staining (Fig. 1d). Fluorescence-activated cell sorting (FACS) analysis of the CD34+/α6 integrin^hi^ bulge SC compartment confirmed progressive depletion of bulge SCs (Fig. 1e; Supplementary Fig. 1b). The initial reduction in the number of bulge SCs was not due to fewer HFs in the skin, as there was no difference in HF density between control and ILK-K5 mice at P21 (Supplementary Fig. 1c). Importantly, expression and localization of Lhx2, Nfatc1 and Sox9, key transcription factors required to establish HFSC fate^17^,^18^,^19^, were not altered. This indicates that ILK is not required to establish HFSC lineage identity (Supplementary Fig. 1d,e).

We next assessed the activation status of the bulge SCs. Upon activation, a subset of bulge SCs transforms into HG cells, divides and generates a pool of transit-amplifying cells (TACs)^20^,^21^. To address whether loss of CD34+ bulge SCs would be associated with SC activation, we stained HFs with antibodies against the SC marker K15 and P-cadherin, a specific marker for TACs^22^,^23^. We observed that P-cadherin staining extended upwards to the bulge where it overlapped with K15 staining in ILK-K5 HFs (Fig. 1f). In addition, K15-positive cells were found in the lower part of the HF corresponding to the hair matrix (Fig. 1f). This suggested that quiescent SCs were reduced, whereas the TAC population expanded to the bulge region of ILK-K5 HFs. To further assess the activation state of the SCs, we analysed proliferation within HFs by applying a short (1 h) bromodeoxyuridine (BrdU) pulse and observed that proliferation within HFs was significantly increased in ILK-K5 mice (Fig. 1g). Furthermore, analyses of long-term ethynyldeoxyuridine (EdU) label retaining as a read-out for the presence of quiescent cells revealed a strong reduction in the quiescent label-retaining cells within ILK-deficient HFs (Fig. 1h) after 10 days of chase, showing that deletion of ILK leads to loss of the quiescent SC pool.

### ILK regulates bulge SCs independent of morphogenesis

**Figure 2:**
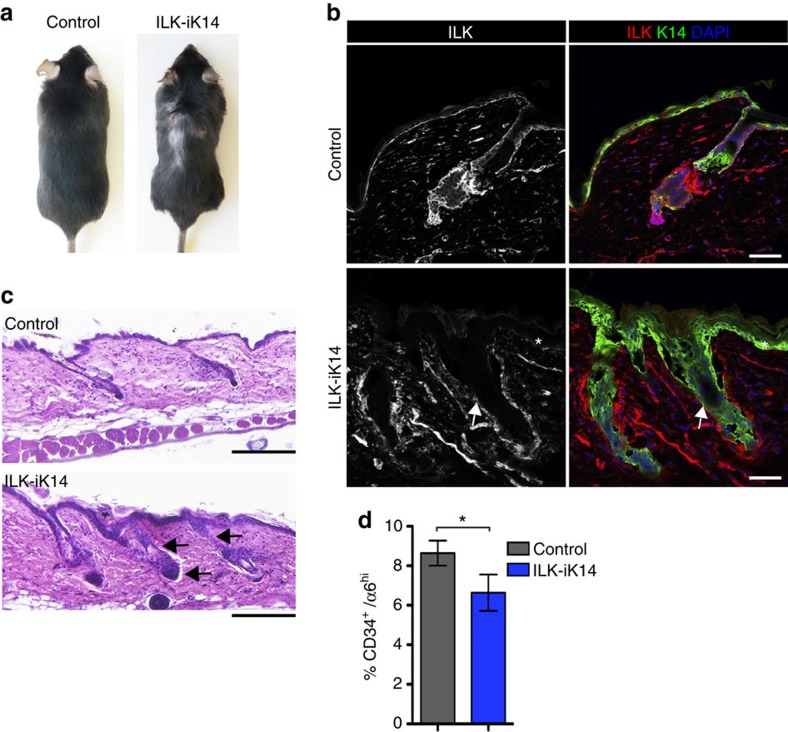
ILK is required to maintain bulge SCs independent of morphogenesis. (**a**) Macroscopic inspection of ILK-iK14 mice after 8 months of doxycycline feeding reveals hair loss and blister-induced wounding. (**b**) Immunofluorescence staining for ILK (red) and K14 (green). ILK staining is not detected within the IFE (asterisk) or HFs (arrow) of ILK-iK14 mice. Scale bars, 50 μm. (**c**) Haematoxylin/eosin staining shows telogen HF morphology in control skin, whereas HFs in ILK-iK14 are enlarged with thickening of both the infundibulum and the outer root sheath (arrows). Scale bar, 200 μm. (**d**) FACS analysis of CD34^+^/α6 integrin^hi^ bulge SCs shows reduction of these cells in ILK-iK14 skin (mean±s.e.m.; *n*=5; *P=0.0313, Wilcoxon matched-pairs test). DAPI, 4,6-diamidino-2-phenylindole.

Given that the bulge niche is established postnatally around P21 when morphogenesis is completed^24^,^25^, we asked whether the loss of quiescent bulge SCs is a consequence of abnormal HF morphogenesis or whether ILK is required for SC maintenance. For this we used a doxycycline-inducible K14-promoter-driven Cre^26^ to delete ILK in the epidermis of adult mice (from here on termed ILK-iK14). We administered doxycycline from P21 onwards and observed that after 8 months of feeding these mice started developing spontaneous, patchy hair loss (Fig. 2a), concomitant with efficient downregulation of ILK protein in the IFE and HFs (Fig. 2b). Histological analyses showed enlarged HFs, with thickened infundibulum and outer root sheath, closely resembling the appearance of ILK-K5 HFs (Fig. 2c). In addition, FACS analysis showed a decrease of CD34+/α6 integrin^hi^ bulge SCs upon deletion of ILK (Fig. 2d). Collectively, these results indicate that ILK is required to maintain quiescence in bulge SCs independent of HF morphogenesis.

### ILK deficiency leads to enhanced bulge SC differentiation

**Figure 3:**
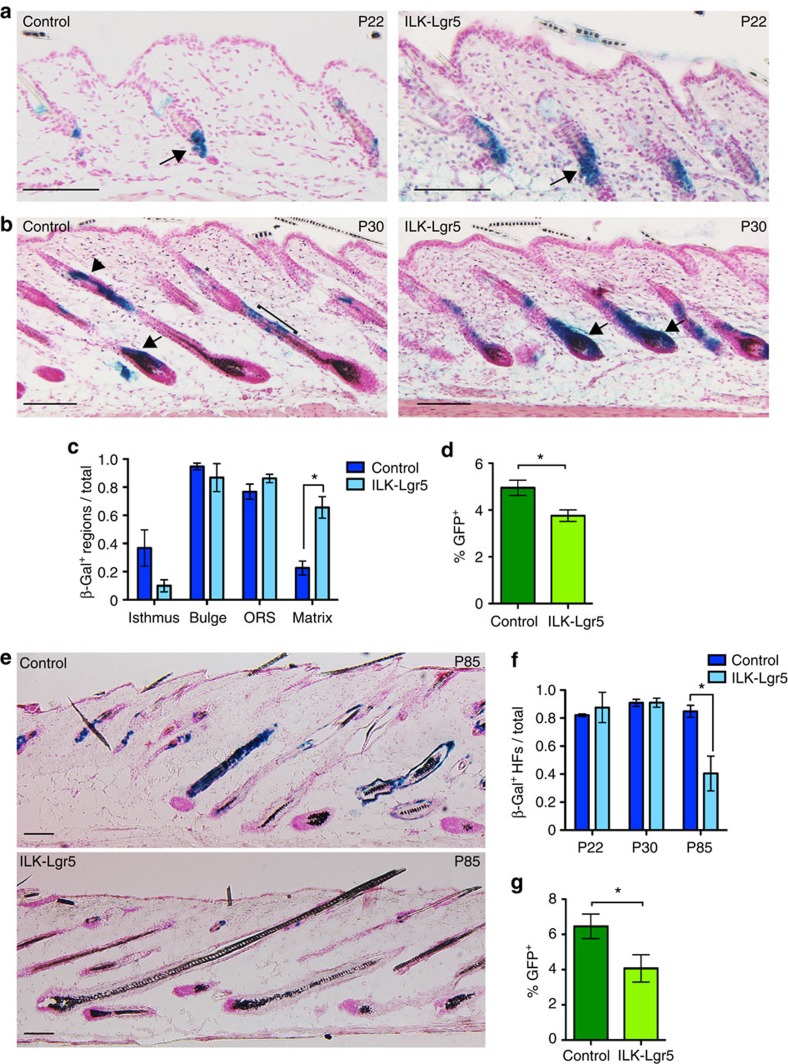
ILK deficiency leads to loss of quiescent SCs through enhanced differentiation. (**a**) Lineage-tracing analysis of β-galactosidase-positive Lgr5+ SC progeny of control and ILK-Lgr5Cre mice in P22 skin, directly after 5 consecutive days of tamoxifen application. β-galactosidase-positive Lgr5 progeny are seen in the bulge and secondary germ regions of HFs (arrows) both in controls and ILK-Lgr5Cre mice. Scale bars, 100 μm. (**b**) After 1 week, (P30) β-galactosidase staining shows Lgr5 progeny in the isthmus (asterisk), bulge (arrowhead), outer root sheath (ORS; bracket) and matrix (arrow) regions in HFs of control skin, whereas in ILK-Lgr5Cre skin the strongest staining is restricted to the matrix of HFs (arrows). Scale bars, 100 μm. (**c**) Quantification of the proportion of P30 HFs that contain β-galactosidase-positive cells within the regions indicated (mean±s.e.m.; *n*=4; *P=0.0159, Mann–Whitney). (**d**) FACS analysis of GFP^+^ Lgr5-expressing cells shows a reduction in this cell population in P30 ILK-Lgr5Cre mice (mean±s.e.m.; *n*=7; *P=0.0013, Student's *t*-test). (**e**) Lineage tracing of Lgr5 progeny at P85 from control and ILK-Lgr5Cre mice shows positive cells throughout HFs of controls, whereas ILK-deleted mice show strongly reduced staining. Scale bars, 100 μm. (**f**) Quantification of the distribution of β-galactosidase staining within P22, P30 and P85 HFs. ILK-Lgr5Cre mice show reduced β-galactosidase staining at P85 (mean±s.e.m.; *n*=4; *P=0.0286, Mann–Whitney). (**g**) FACS analysis of GFP^+^ Lgr5-expressing cells shows a reduction in this cell population in P85 ILK-Lgr5Cre mice (mean±s.e.m.; *n*=4; *P=0.0381, Mann–Whitney).

We next sought to trace the fate of the aberrantly activated ILK-deficient SCs *in vivo*. To this end, we crossed ILK^fl/fl^ mice with Lgr5-EGFP-Ires-Cre^ERT2^ mice^27^ that were additionally carrying the Rosa26R-LacZ-Cre-reporter allele^28^ (from here on termed ILK-Lgr5 mice). Administration of tamoxifen at P17 (shortly before onset of telogen) to activate Cre resulted in efficient deletion of ILK specifically within HFs (Supplementary Fig. 2a). When HFs are in telogen, Lgr5 is exclusively expressed in SCs located in the lower bulge and in HG cells. Upon entry into anagen, the progeny of these cells contribute to the lower non-permanent part of the HF, including the cells populating the hair matrix, which no longer express Lgr5. In addition, in a process dependent on active cycling, progeny of Lgr5 cells can repopulate the bulge^29^. We injected mice from P17 onwards and observed that in the first telogen phase at P21/22, Lgr5-expressing cells were labelled by β-galactosidase and localized to the bulge and HG regions both in control and ILK-Lgr5 HFs (Fig. 3a).

When the HFs entered the subsequent anagen phase at P30, control mice showed β-galactosidase-positive Lgr5 progeny in the isthmus and bulge regions as well as in the non-permanent part of the HF, including a subset of the hair matrix cells (Fig. 3b,c). Strikingly, the frequency of β-galactosidase staining was significantly increased in the hair matrix of ILK-Lgr5 HFs, accompanied by a more subtle decrease in β-galactosidase staining in the isthmus region (Fig. 3b,c), indicating that deletion of ILK induces Lgr5-positive bulge SCs to leave the bulge and differentiate into hair matrix cells. This observation was strengthened by FACS analysis showing reduced numbers of GFP^+^ Lgr5-expressing SCs at this stage (Fig. 3d; Supplementary Fig. 2b).

Analyses of HFs in the second anagen phase post tamoxifen induction (P85) revealed that the overall numbers of β-galactosidase-positive cells found within HFs were significantly reduced in ILK-Lgr5 mice (Fig. 3e,f), confirming that Lgr5 progeny had been lost due to their preferential localization in the non-permanent part of the HFs in the preceding hair cycle. Consistent with this, the amount of GFP^+^ Lgr5-expressing cells was further diminished (Fig. 3g). Together these data show that upon loss of ILK, Lgr5-positive HFSCs are aberrantly activated and mobilized to differentiate into hair matrix cells during anagen, whereas repopulation of the bulge by Lgr5 progeny in the subsequent telogen fails to occur. This results in progressive depletion of Lgr5+ SCs.

### ILK is required to remodel the ECM around the bulge SC niche

**Figure 4:**
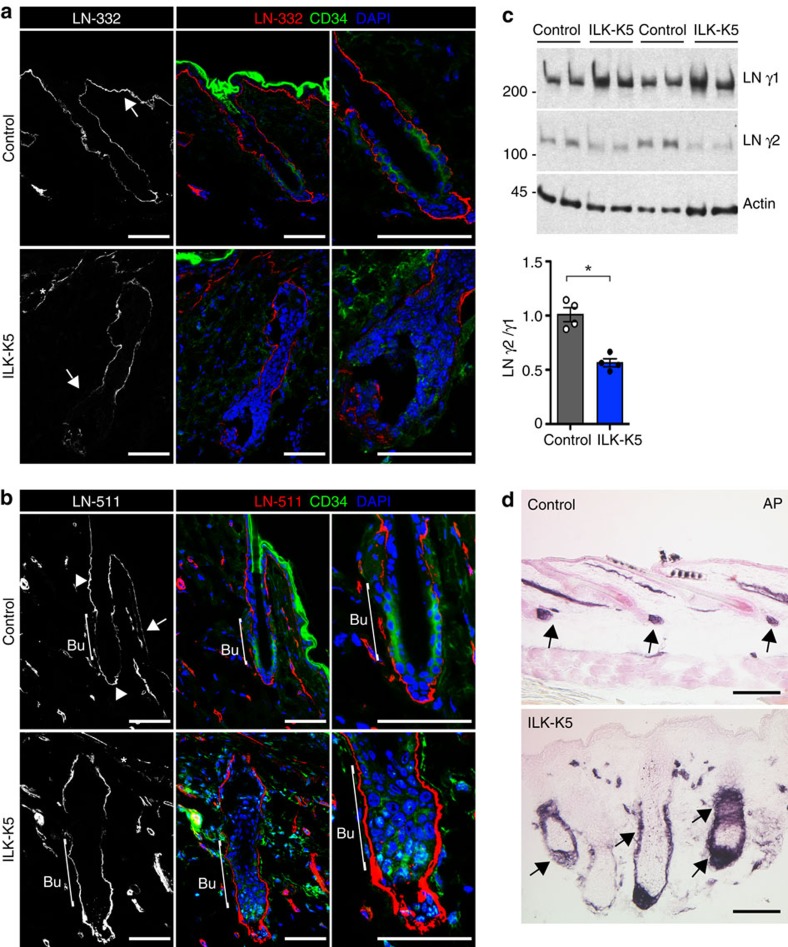
ILK is required to remodel the ECM around the bulge SC niche. (**a**) Immunofluorescence staining for LN-332 (red) and CD34 (green) from P21 HFs. LN-332 staining shows higher intensity beneath IFE (arrow) than around HFs in control skin (upper panel). Note fragmentation of LN-332 staining along the IFE (asterisk) and decreased LN-332 around the lower part of the HF, including the HG (arrow) in ILK-K5 skin (lower panel). Scale bars, 50 μm. (**b**) Immunofluorescence staining for LN-511 (red) and CD34 (green) from P21 HFs. LN-511 staining shows highest intensity at the isthmus region and around HG (arrowheads). Only faint staining is observed beneath the IFE (arrow) and around bulge (bracket) in control skin (upper panel). Note fragmentation of LN-511 staining along the IFE (asterisk) and high intensity around bulge and HG (bracket) in ILK-K5 skin (lower panel). Scale bars, 50 μm. (**c**) Western blot analysis of LN γ1 and γ2 chains in skin extracts. Actin is used as a loading control. ILK-K5 skin shows increased levels of γ1 and decreased levels of γ2, resulting in a twofold decrease in γ2 to γ1 ratio. Lower panel shows quantifications of mean band intensities (mean±s.d.; *n*=4; *P=0.0286, Mann–Whitney). For full scans of western blots, see Supplementary Fig. 7. (**d**) Alkaline phosphatase (AP) staining to detect dermal papilla (DP) cells in P21 skin. In control skin, the DP is found attached to the base of each HF (arrows; upper panel). In ILK-K5 skin AP-positive cell population is increased and surrounds the entire lower region of the HFs (arrows; lower panel). Scale bars, 200 μm. DAPI, 4,6-diamidino-2-phenylindole.

As we had previously observed that ILK is essential for ECM deposition^13^, we hypothesized that changes in the ECM microenvironment could play a role in the aberrant activation of SCs in ILK-deficient HFs. To analyse this, we performed immunofluorescence analyses of two central BM components along the HFs, laminin (LN)-332 and LN-511 (ref. 30). Analysis of control mice showed that the LNs formed linear inverse gradients between the IFE and HFs. LN-332 expression was higher in the IFE compared with the deeper portions of the HF (Fig. 4a). In contrast, LN-511 was less abundant beneath the IFE, and the staining intensified around the HFs with the strongest signal at the isthmus region and around the HG (Fig. 4b). However, the bulge region showed weak LN-511 staining (Fig. 4b). In contrast, and as previously reported^15^, the BM beneath the IFE was fragmented in the ILK-K5 skin, as shown with both LN-332 and LN-511 staining (Fig. 4a,b). Interestingly, the BM was found to be relatively intact around the HF isthmus. Strikingly, LN-511 staining was highly enriched around the putative bulge region and around the HG of ILK-deficient HFs (Fig. 4b). In contrast, LN-332 staining was reduced around the lower portion of the HF including the HG (Fig. 4a). Western blot analysis of skin extracts with antibodies against γ1 and γ2 chains of LN-511 and LN-332, respectively, indicated that the amounts of LN γ2 chain were decreased and those of the LN γ1 chain were increased in ILK-K5 skin extracts compared with controls (Fig. 4c), demonstrating that the LN-332/LN-511 ratio is markedly altered in the skin BM of ILK-K5 mice.

We next asked whether the altered BM remodelling would be associated with changes in the dermal papilla (DP), a specialized cluster of mesenchymal cells that attaches to the HF BM and regulates HFSC activation^31^,^32^. Analysis of the DP at P21 showed that DP was attached to the distal tip of the HG in control telogen HFs (Fig. 4d), as expected. Strikingly, the DP of ILK-K5 HFs encapsulated the entire lower part of the HF, including the putative bulge (Fig. 4d). The alteration in DP architecture was already seen during the anagen stage at P14, excluding that the effect would be due to a difference in hair cycle stage (Supplementary Fig. 3a). Importantly, both the defects in BM composition and DP architecture were also seen when ILK was deleted in adult mice, demonstrating that they were not due to impaired HF morphogenesis (Supplementary Fig. 3b–d). These data indicate that ILK regulates the architecture of the bulge SC BM niche, as well as the crosstalk between the bulge SCs and the DP.

### ILK regulates SC fate-determining pathways

**Figure 5:**
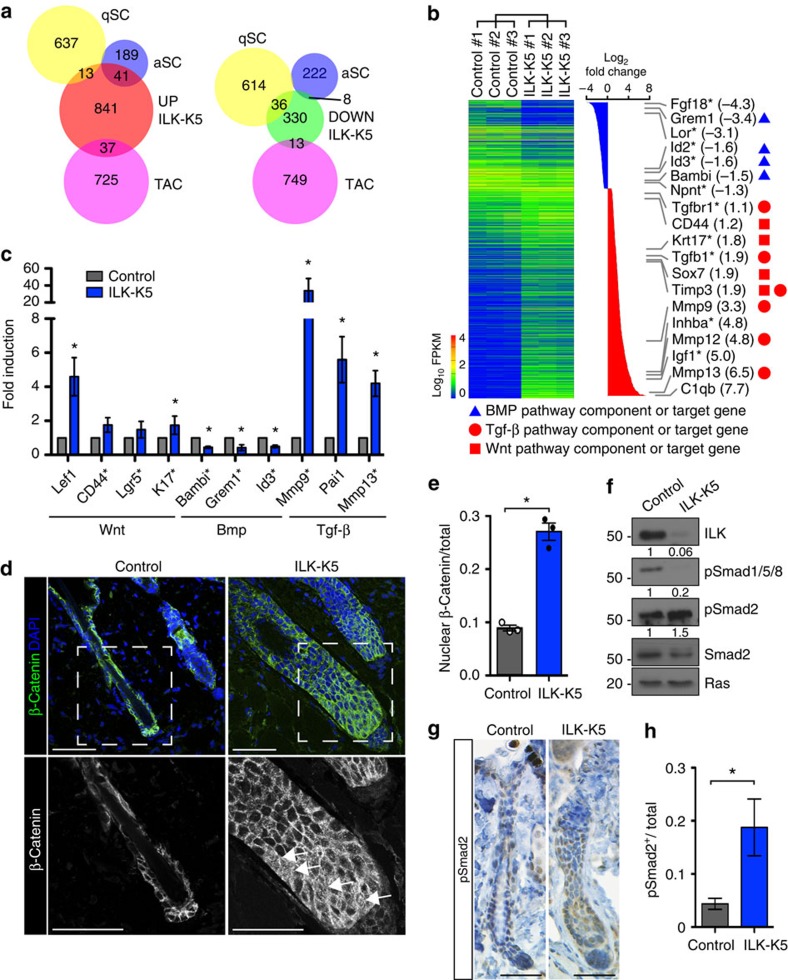
Deregulation of key SC fate-determining pathways upon deletion of ILK. (**a**) Schematic representation of RNA-seq profiling of transcripts up- or downregulated (padj<0.05) in purified populations of ILK-K5 bulge SCs compared with controls (‘UP ILK-K5' and ‘DOWN ILK-K5', respectively), compared with published gene expression signatures of quiescent bulge SCs (qSCs), activated bulge SCs (aSCs) and TACs. Note overlap of upregulated genes with aSC and TAC signatures, and overlap of downregulated genes with qSC signature. (**b**) Summary of transcriptional profiling of purified bulge SCs from ILK-K5 and control skin. Significantly regulated genes are listed on the right side. Note the genes involved in BMP (blue triangle), Tgf-β (red circle) and Wnt (red square) signalling. Other genes implicated in HFSC biology are marked with an asterisk. (**c**) qRT–PCR validation of RNA-seq data from independently derived bulge SC RNA samples. Note upregulation of Wnt and Tgf-β pathway target genes and downregulation of BMP pathway target genes. Genes that were found significantly regulated in the RNA-seq data are marked with an asterisk (mean±s.e.m.; *n*=4; *P=0.0211, Mann–Whitney). (**d**) Immunofluorescence staining for β-Catenin (in green) from P21 HFs. Lower panel represents blow up of area marked with a white rectangle. Note increased nuclear localization of β-Catenin in ILK-K5 HFs (arrows). Scale bars, 50 μm. (**e**) Quantification of nuclear β-Catenin staining (mean±s.e.m.; *n*=3; *P=0.05, Mann–Whitney). (**f**) Western blot analysis from P21 epidermal lysates shows increased phosphorylation of Smad2 and decreased phosphorylation of Smad1/5/8 in ILK-K5 skin. Quantifications represent mean band intensities from three independent experiments. For full scans of western blots, see Supplementary Fig. 7. (**g**) Immunohistochemical staining for pSmad2 from P21 HFs. Note increased pSmad2 staining (in brown) in ILK-K5 HFs. Scale bars, 30 μm. (**h**) Quantification of pSmad2 staining (mean±s.e.m.; *n*=5; *P=0.0297, Student's *t*-test). DAPI, 4,6-diamidino-2-phenylindole; ANOVA, analysis of variance.

We next asked whether the altered architecture of the lower HF and the SC niche would perturb signalling crosstalk between the HF and the DP, thereby affecting bulge SC activation. To assess the signalling status within ILK-deficient bulge SCs, we sorted CD34+/α6 integrin^hi^ SCs and analysed their transcriptomes using RNA-sequencing (RNA-seq). We found 932 genes to be significantly upregulated and 387 genes to be downregulated padj ≤0.05; Fig. 5a; Supplementary Data 1). To analyse whether the altered gene expression profile would reflect the enhanced activation and differentiation of the bulge SCs observed in the lineage-tracing experiments, we compared the gene expression signature of ILK-deficient bulge SCs with published signatures of quiescent and activated bulge SCs as well as TACs^33^. Strikingly, a significant proportion of the upregulated genes were signature genes of activated bulge SCs and TACs, whereas downregulated genes were over-represented in the quiescent bulge SC signature (Fig. 5a). Gene ontology (GO) term analysis provided further insights into biological processes that were altered in ILK-deficient bulge SCs. Epithelium development and cell adhesion surfaced among top GO terms in the group of downregulated genes, whereas immune response, response to wounding and cell activation were enriched in the upregulated genes (Supplementary Fig. 4a).

The quiescent SC state is characterized by low activity of the Wnt and Tgf-β2 pathways and high Bmp activity^2^. Interestingly, Kyoto Encyclopedia of Genes and Genomes (KEGG) pathway analysis revealed that Bmp pathway genes were the most significantly over-represented gene group in the downregulated genes (*P*=0.0065, Fisher's exact test), whereas the cytokine–cytokine receptor pathway, including the Tgf-β pathway, was most significantly over-represented in the upregulated gene set (*P*=1.12 × 10^−14^, Fisher's exact test; Fig. 5b; Supplementary Fig. 4a). Also multiple target genes of the Wnt pathway were found significantly upregulated in ILK-K5 bulge SCs (padj ≤0.05; Fig. 5b).

To validate the results of the RNA-seq analysis, we further analysed gene expression of independently isolated bulge SCs using reverse transcription (RT)–quantitative (q)PCR. In agreement with the RNA-seq data, the gene expression signature of ILK-deficient bulge SCs showed increased expression of Wnt and Tgf-β2 pathway target genes (*Lef1*, *CD44*, *Lgr5* and *K17* for Wnt and *Mmp9*, *Pai1*, *Mmp13* for Tgf-β2), whereas Bmp pathway target genes (*Bambi*, *Grem1* and *Id3*) were downregulated (Fig. 5c).

The changes in target gene expression were paralleled by increased nuclear localization of β-catenin, in particular, within the enlarged HG at P21 (Fig. 5d,e) as well as increased staining of Lef1 that extended to the K15-positive compartment within the bulge (Supplementary Fig. 4b), indicative of increased Wnt signalling. Furthermore, we observed downregulation of Smad1/5/8 phosphorylation as evidence for reduced Bmp activity (Fig. 5f) and upregulation of Smad2 phosphorylation indicative of enhanced Tgf-β signalling (Fig. 5f–h). Taken together, the data demonstrate that deletion of ILK results in a shift towards an activated SC phenotype characterized by upregulated Tgf-β2 and Wnt/β-catenin signalling and supressed BMP signalling at P21, when SCs are normally quiescent.

To assess whether the observed alterations in the signalling status of the bulge SCs were secondary to the fact that ILK-K5 HFs were not properly entering telogen, we performed gene expression analyses at P14, when both controls and ILK-K5 HFs were phenotypically in late anagen (Supplementary Fig. 4c). As the CD34+ bulge SC niche is not yet established at this stage^34^, we isolated α6 integrin+ EPCs that contain progenitor/SCs from both HFs and IFE for gene expression analyses. RT–qPCR analyses of P14 EPCs revealed that Wnt and Tgf-β pathway target genes were upregulated already during the anagen stage (Supplementary Fig. 4d), indicating that the changes did not arise from a difference in the hair cycle phase. Interestingly, Bmp pathway targets were unaltered at this point (Supplementary Fig. 4d), suggesting that changes in Bmp signalling were not the primary cause of the phenotype. Interestingly, at the onset of anagen at P7, no major differences in Wnt target gene expression were found, with the exception of upregulated Lgr5 expression (Supplementary Fig. 4e), consistent with previous results showing no obvious changes in Wnt reporter activity during anagen^15^. Collectively, the data indicate that HFs lacking ILK fail to downregulate Wnt/β-catenin and Tgf-β2 pathways during the anagen-to-catagen transition, leading to persistent SC activation and subsequent failure to enter telogen.

### BM composition regulates SC quiescence and activation

**Figure 6:**
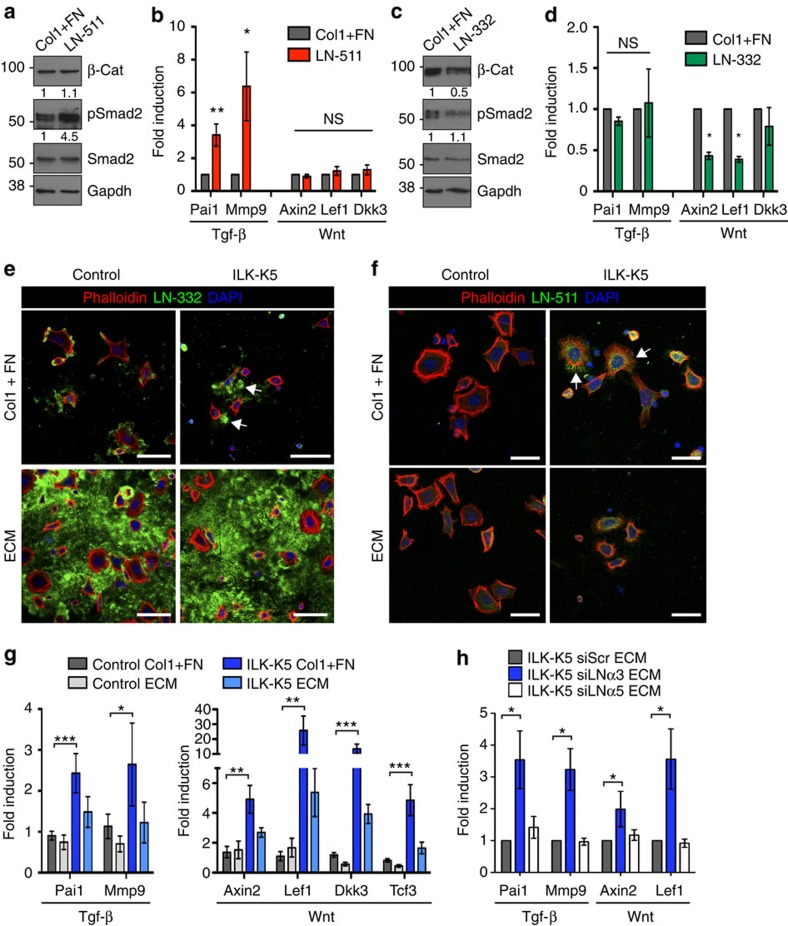
The composition of the BM regulates SC quiescence and activation. (**a**) Western blot analysis of freshly isolated epidermal progenitor cells (EPCs) plated on LN-511 or a collagen I/fibronectin mixture (Col1+FN) as control. Cells on LN-511 show increased Smad2 phosphorylation, whereas β-Catenin (β-Cat) is unchanged. Quantifications represent mean band intensities from three independent experiments. (**b**) RT–qPCR analysis of Wnt/β-Catenin and Tgf-β target gene expression shows selective upregulation of Tgf-β target genes in cells adhering to LN-511 (mean±s.e.m.; *n*=5; *P=0.0312 **P=0.0075, Mann–Whitney). (**c**) Western blot analysis of EPCs plated on LN-332 or Col1+FN as control. Cells on LN-332 show decreased β-Catenin levels, whereas Smad2 phosphorylation in unchanged. Quantifications represent mean band intensities from three independent experiments. (**d**) Analysis of Wnt and Tgf-β pathway target gene expression shows selective downregulation of Wnt/β-catenin target genes in cells adhering to LN-332 (mean±s.e.m.; *n*=4; *P=0.0211, Mann–Whitney). (**e**) Immunofluorescence analyses of EPCs adhering to Col1+FN (upper panel) or preassembled ECM (lower panel). Control cells deposit LN-332 matrix on which they adhere to (in green), whereas EPCs from ILK-K5 skin deposit LN-332 aggregates (arrows). Preassembled ECM contains large amounts of LN-332 that supports adhesion in both control and ILK-K5 EPCs (lower panels). Phalloidin (red) was used to counterstain adhering cells. Scale bars, 35 μm. (**f**) Immunofluorescence analyses of EPCs plated on Col1+FN (upper panel) or preformed ECM (lower panel). On Col1+FN, control cells deposit very low levels of LN-511 (in green), whereas ILK-K5 EPCs deposit larger amounts (arrows). Preassembled ECM contains very low levels of LN-511 (lower panels). Phalloidin (red) was used to counterstain adhering cells. Scale bars, 25 μm. (**g**) Adhesion of EPCs from ILK-K5 skin on preassembled wild-type ECM restores Wnt and Tgf-β pathway target gene expression to the level of control cells (mean±s.e.m.; *n*=7; ***P<0.0003, **P<0.0048, *P=0.0437, analysis of variance (ANOVA)/Bonferroni). (**h**) ECM where LN-332 has been depleted (siLNα3) fails to downregulate Wnt and Tgf-β pathway target gene expression in ILK-K5 EPCs, whereas depletion of LN-511 (siLNα5) has no effect (mean±s.e.m.; *n*=3; *P<0.05, ANOVA/Bonferroni). For full scans of all western blots, see Supplementary Fig. 7. DAPI, 4,6-diamidino-2-phenylindole; NS, not significant.

We next asked whether the observed changes in the bulge SC niche of ILK-K5 HFs could directly contribute to the aberrant activation of SCs. We first tested whether the inverse gradients of LN-332 and LN-511 could differentially regulate SC activation. We isolated EPCs from wild-type mice, plated them directly on recombinant LNs and analysed Wnt and Tgf-β signalling. A mixture of two other β1 integrin ligands, type I collagen and fibronectin, were used as controls to exclude effects from altered adhesion or integrin usage. Adhesion of EPCs on LN-511 induced phosphorylation of Smad2 (Fig. 6a) as well as upregulation of Tgf-β target genes (Fig. 6b), whereas it did not affect β-catenin stability or Wnt target gene expression (Fig. 6a,b). In contrast, adhesion of EPCs on LN-332 suppressed β-catenin levels (Fig. 6c), and downregulated the expression of Wnt target genes (Fig. 6d), whereas it did not affect Tgf-β signalling (Fig. 6c,d). Together these results indicate that LN-511, present at low levels around the bulge and at higher levels around the HG/TACs, promotes Tgf-β signalling, whereas LN-332, highly expressed along the IFE and to a lesser extent along the upper regions of HFs, suppresses Wnt/β-catenin signalling.

To address whether the altered LN-332/LN-511 ratio in ILK-K5 skin could cause increased Tgf-β2 and Wnt signalling, we performed *in vitro* rescue experiments where we substituted the ECM of ILK-deficient EPCs by that deposited by wild-type keratinocytes. In contrast to control cells, ILK-deficient EPCs were unable to deposit a proper LN-332 pericellular matrix (Fig. 6e). Instead they deposited abnormal aggregates of LN-332 that did not support adhesion, as cells were found to displace themselves from the LN-332 aggregates (Fig. 6e). When plated on preassembled wild-type ECM that was abundant in LN-332, ILK-deficient EPCs readily adhered to this matrix (Fig. 6e). Analysis of LN-511 revealed that control cells deposited very little LN-511, whereas ILK-deficient EPCs deposited and adhered to LN-511 (Fig. 6f). Consistent with the low abundance of LN-511 pericellular matrix in control cells, very little LN-511 was detected in the preassembled ECM (Fig. 6f).

ILK-deficient EPCs maintained the molecular signature of high Tgf-β and Wnt target gene expression seen *in vivo* also when placed into culture (Fig. 6g). Strikingly, the altered gene expression was completely restored when ILK-deficient cells were plated on wild-type preassembled ECM (Fig. 6g). To confirm that the rescue in signalling was induced by correcting the LN-332/LN-511 ratio, we depleted these LNs from the ECM-producing cells using short interfering RNAs (siRNAs) (Supplementary Fig. 5a,b). Interestingly, depletion of LN-332 induced a slight increase in LN-511 deposition into the ECM of wild-type keratinocytes (Supplementary Fig. 5a,b), resembling the constitution of the BM around the ILK-deficient HFs (Fig. 4a–c). Importantly, ILK-K5 EPCs plated on the LN-332-depleted ECM where LN-511 was now relatively more abundant failed to downregulate Wnt and Tgf-β signalling (Fig. 6h). As expected, further depletion of the low LN-511 levels did not significantly affect the ability of the ECM to rescue the altered Wnt and Tgf-β signalling activities in ILK-K5 EPCs (Fig. 6h). We conclude that the precise ratio between LN-332 and LN-511 adjusts activities of key signalling pathways that determine SC activation within the bulge niche. This ratio is disturbed in the absence of ILK, resulting in aberrant SC activation and subsequent SC exhaustion.

### SC activation promotes replicative stress and skin carcinogenesis

**Figure 7:**
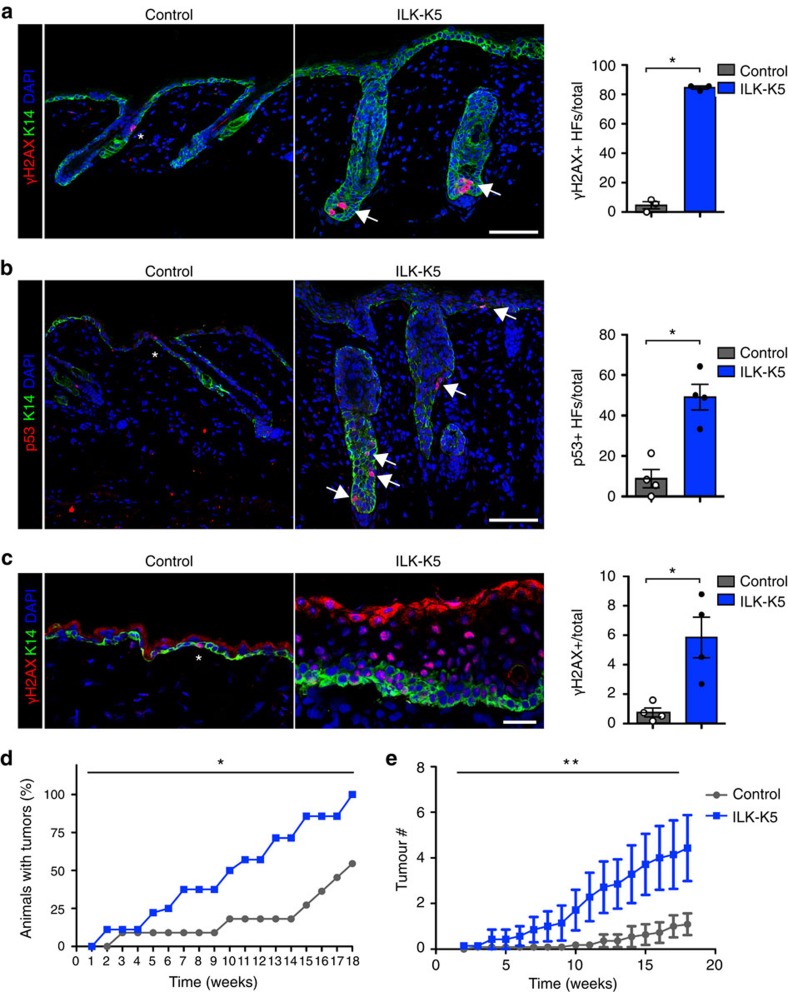
SC activation promotes replicative stress and skin carcinogenesis. (**a**) Immmunofluorescence staining for γH2AX (red) and K14 (green) from P21 skin. Control mice rarely show γH2AX-positive cells (asterisk), whereas ILK-K5 HFs show clusters of cells with pan-nuclear γH2AX within HFs (arrows). Scale bars, 50 μm. Right panel shows quantification of HFs containing more than two γH2AX-positive cells (mean±s.e.m.; *n*=3; *P=0.0383, Mann–Whitney). (**b**) Staining for p53 (red) and K14 (green) from P21 skin. In contrast to control mice that show only solitary p53-positive cells (asterisk), ILK-K5 mice frequently show p53-positive cells within HFs and IFE (arrows). Scale bars, 50 μm. Right panel shows quantification of HFs containing more than two p53-positive cells (mean±s.e.m.; *n*=4; *P=0.0286, Mann–Whitney). (**c**) Staining for γH2AX (red) and K14 (green) from P57 skin. Control mice show only solitary γH2AX-positive cells (asterisk) within the IFE, whereas ILK-K5 IFE shows abundant pan-nuclear γH2AX staining. Scale bars, 50 μm. Right panel shows quantification of γH2AX-positive cells per total cells within the IFE (mean±s.e.m.; *n*=4; *P=0.0286, Mann–Whitney). (**d**) Tumour incidence of control and ILK-K5 mice treated twice with DMBA followed by 18 weeks of biweekly TPA treatment. ILK-K5 mice show increased tumour incidence (*n*=11/11; *P=0.0209, *χ*^2^-test). (**e**) Tumour multiplicity in affected control and ILK-K5 mice during 18 weeks of biweekly TPA treatment. ILK-K5 mice show increased tumour multiplicity (mean±s.e.m.; *n*=11/11; **P<0.01, two-way analysis of variance). DAPI, 4,6-diamidino-2-phenylindole.

We next sought to identify long-term consequences of the aberrant SC activation in ILK-deficient skin. Quiescent bulge SCs are more resistant to DNA damage than their progeny due to a more efficient DNA damage response, whereas SC activation can promote the generation of tumour-initiating cells^35^. In addition, quiescence prevents replicative stress and protects SCs from DNA damage in hematopoietic SCs^36^. We thus hypothesized that enhanced activation would expose ILK-deficient bulge SCs to replicative stress and thereby to DNA damage. Analysis of pan-nuclear γH2AX staining indicative for replicative stress^37^, showed a marked increase in ILK-K5 epidermis at P21 (Fig. 7a). The replicative stress was further confirmed by elevated levels of p53 (Fig. 7b).

We next asked whether replicative stress in ILK-K5 skin was restricted to the phase of aberrant bulge SC activation and depletion. To this end, we analysed mice at P57, when bulge SCs were largely depleted in ILK-K5 mice, and observed accumulation of pan-nuclear γH2AX staining within the ILK-deficient IFE (Fig. 7c). The IFE of ILK-K5 mice showed hyperproliferation at this stage (Fig. 7c). As it was recently shown that repression of Wnt/β-catenin signalling in the IFE is required to maintain normal levels of proliferation under homeostatic conditions^38^,^39^, we asked whether Wnt signalling would be upregulated within the IFE at P57. Analyses of Wnt target gene expression from sorted ILK-K5 EPCs showed that Wnt target genes were still upregulated at P57, despite low abundance of HFs (Supplementary Fig. 6a).

As we observed increased replicative stress and subsequent accumulation of DNA damage in ILK-deficient mice, we next investigated whether this would predispose the mice to skin carcinogenesis. Using the chemical induced multistage skin carcinogenesis protocol^40^, we observed that ILK-K5 mice displayed a significant increase in tumour incidence (Fig. 7d; Supplementary Fig. 6b). The tumour multiplicity was also significantly increased (Fig. 7e). The morphology of the papillomas was comparable, and the tumour size, proliferation and apoptosis rates within them were not altered (Supplementary Fig. 6c–f). This indicated that ILK does not significantly regulate the behaviour of the tumour cells after transformation. Studies on tumour progression were not possible due to deteriorating skin health condition of ILK-K5 mice. Taken together, loss of ILK leads to upregulation of Wnt signalling in HFs and in the IFE and this increased activation is associated with increased DNA damage, predisposing the tissue to malignant transformation.

## Discussion

In the present study we show that ILK is required for the maintenance of quiescent bulge SCs through remodelling of the ECM niche. We further show that the precise ratio of LN-511 and LN-332 regulates core SC fate-determining signalling pathways and that this ratio is disturbed in the absence of ILK, leading to aberrant SC activation and failure to re-establish quiescence. In the long run, the enhanced activation predisposes ILK-deficient cells to replicative stress and malignant transformation.

Adult SCs fuel tissue renewal, repair and remodelling in mature organs. By tuning their proliferation rate to match the changing needs of their resident tissues, SCs maintain organ form and function. Their ability to sense and respond to tissue needs derives, in large part, from the intimate association of SCs with their niche^1^. Because of their complexity and dynamic variation, niches of mammalian adult SCs are still poorly characterized. The skin epithelium, due to its geometric and well-understood architecture, offers particular potential for shedding light on the role of the ECM niche in SC regulation. We have previously shown that ILK is critical for remodelling the ECM, due to its essential role in actin organization and in generating force through β1 integrins^13^, making ILK-deficient SCs an ideal model to address this question. Our current data show that ILK-mediated establishment of inverse gradients of the β1-integrin ligands LN-511 and LN-332 within the IFE and HFs is central for maintenance of the quiescent bulge SC pool.

**Figure 8:**
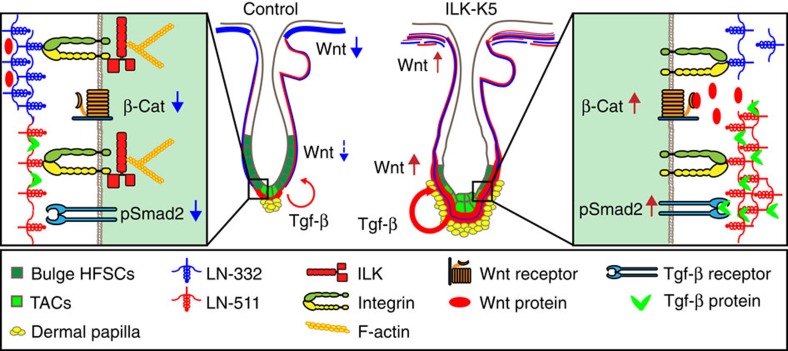
Model of ILK-mediated signalling in the epidermis and HFs. LN-332 (in blue) and LN-511 (in red) form inverse gradients within the epidermal basement membrane. LN-332 is abundant beneath the interfollicular epidermis, whereas LN-511 is most abundant at the lower part of the hair follicle, surrounding the transit-amplifying cells (TACs). LN-332 suppresses Wnt signalling, whereas LN-511 promotes Tgf-β signalling. Deletion of ILK leads to fragmentation of the LN-332-BM beneath the IFE and around the lower part of the HF, whereas LN-511 levels around the hair follicle increase, possibly due to compensatory deposition by the DP cells. This relative increase in LN-511 levels leads to increased Wnt and Tgf-β signalling, resulting in aberrant bulge SC activation and their subsequent depletion due to enhanced differentiation.

In human and in mice, absence of LN-332 results in extensive skin blistering and lethality few days after birth, highlighting its importance for epidermal architecture, but precluding analysis of HF cycling and postnatal IFE homeostasis^41^. In contrast, LN-511 that is present in highest levels around the HG is clearly needed for HF downgrowth^42^,^43^. Interestingly, LN-511 is downregulated during anagen-to-catagen transition, and this downregulation is required for catagen progression^30^. Consistently, exogenous LN-511 enhances the growth of human HFs, whereas addition of LN-332 antagonizes this effect^44^, indicating that LN-511 and LN-332 play opposing roles in HF growth regulation. Our findings that LN-511 enhances Tgf-β signalling, which makes HFSC more receptive for activating signals, whereas LN-332 suppresses Wnt signalling that is required for HFSC differentiation^2^, provide a molecular mechanism for these observations and explain why ILK-deficient HFs fail to re-establish SC quiescence and to enter telogen. We summarize these findings in a model in Fig. 8.

How LN-511 and LN-332 regulate these specific signalling pathways remains an exciting open question. Compared with LN-332, LN-511 shows higher affinity to heparan sulfate proteoglycans^45^,^46^. Moreover, enzymatic removal of the proteoglycan-binding domain of LN-332 is required for integration of LN-332 to the BM network^8^. As heparan sulfate proteoglycans are important regulators of growth factor signalling, altered LN-332/LN-511 ratios could result in differential deposition and bioavailability of Wnt and/or Tgf-β2, or directly affect downstream signalling through modulating transmembrane heparan sulfate proteoglycans such as syndecans^47^. Alternatively, binding of β1 integrins to different LN isoforms might lead to assembly of differential integrin–adhesion complexes^48^, thereby affecting signalling crosstalk with growth factor receptors.

The epidermis consists of multiple SC niches that harbour distinct pools of SCs and regulatory niche cells. Despite this complexity, a unifying feature of the different SC populations is that they adhere basally to BMs that integrate them into their specific tissue architecture^49^, raising the possibility that structural, BM-derived signals coordinate the various SC niches. Intriguingly, when restricted by tissue architecture, the specific SC pools within the epidermis remain strictly compartmentalized, and the pilosebaceous unit is maintained independent of the IFE in the absence of wounding^50^,^51^,^52^. However, upon removal from tissue and subsequent transplantation, the specialized SCs exhibit broader potency in their new microenvironment. For example, transplanted bulge SCs generate not only HFs, but also IFE and sebaceous glands^53^,^54^. This strongly indicates that differences in the molecular composition of the HF and IFE niches guide SC lineage progression. Our finding that LN-332 that is enriched in the IFE suppresses Wnt signalling, could explain the ability of the IFE niche to drive HFSCs towards IFE fate. The question remains how ILK differentially regulates LN-511 and LN-332 deposition. ILK is essential for the force-transducing function of β1 integrins that bind both LN-511 and LN-332 with high affinity^55^. Interestingly, dystroglycans that are ECM-assembling receptors linked to the actin cytoskeleton, do not bind efficiently to LN-332 but have high affinity to LN-511 (refs 56, 57). Dystroglycans do not require ILK for their function and could compensate for the loss of β1-integrin-ILK function selectively in LN-511 deposition, leading to an imbalance in the ratio of LN-511 and LN-332. Alternatively, as dermal fibroblasts have been shown to contribute to BM assembly at the dermo-epidermal junction^9^,^58^,^59^, excess LN-511 could be derived from the mesenchymal cells of the DP that abnormally encapsulate the ILK-deficient HFs.

Previous studies from us and others have established an essential role for ILK in cell migration^11^. This is consistent with the reported pro-metastatic function of ILK^60^. There are, however, very few mechanistic *in vivo* studies on the role of ILK in cancer. The mouse mammary tumour virus (MMTV) oncogene-induced breast cancer model has been used to address the role of ILK in cancer progression. In this study, ILK expression was shown to promote tumour progression^61^. Our current results show that deletion of ILK leads to hyperproliferation of the IFE, associated with upregulation of Wnt target genes and increased replicative stress. These changes predispose ILK-deficient epidermis to skin carcinogenesis, suggesting that ILK might play a protective role in cancer initiation by maintaining tissue architecture. This is distinct from the oncogene overexpression model of MMTV, as in the latter case only events that occur downstream of oncogene-induced transformation can be explored. To our knowledge, the current study is the first genetic study to assess the role of ILK in the very early steps of cancer initiation, highlighting the importance of using different tumour models to address the various roles of ILK in cancer.

In summary, our study delineates how reciprocal interaction between SCs and their niches regulate SC fate and highlights the role of tissue architecture in coordinating cellular behaviours and protecting it from malignant transformation.

## Methods

### Animal experiments

To generate an epidermis-specific deletion of ILK, ILK^fl/fl^ mice were crossed with mice expressing Cre under the K5 promoter (ILK-K5 (ref. 15)). As no phenotypic differences were observed among ILK^+/+^, ILK^fl/fl^ and ILK^fl/+^-K5-Cre mice, as all showed normal skin and HFs, ILK^fl/+^-K5-Cre mice were chosen as controls to exclude effects from Cre expression. To achieve inducible epidermal deletion of ILK, ILK^fl/fl^ mice were crossed with K14 rTA-tetOCre mice^26^. Cre expression was induced by administering doxycycline in chow (1 g kg^−1^, Ssniff GmbH) for 8 months starting at 3 weeks of age. ILK^fl/+^-K14 rTA-tetOCre and ILK^fl/fl^-tetOCre mice that were phenotypically indistinguishable were used as controls.

For lineage-tracing experiments ILK^fl/fl^ mice were crossed with Lgr5-EGFP-Ires-CreERT2 mice^27^ and Rosa26R-LacZ Cre reporter mice^28^. Cre recombinase was activated by injecting 1 mg tamoxifen dissolved in 100 μl corn oil for 5 consecutive days. Lgr5-EGFP-Ires-CreERT2-ILK^fl/+^ or Lgr5-EGFP-Ires-CreERT2-ILK^+/+^ mice that were phenotypically indistinguishable were used as controls. All mouse lines were in a C57Bl6-129/Sv background.

For all *in vivo* experiments, the controls were littermates of ILK-deficient mice. Genders were distributed randomly between genotypes. Mice were housed in a special pathogen-free mouse facility. All animal experiments were performed according to institutional guidelines and animal licence of the State Office North Rhine-Westphalia, Germany.

### *In vivo* proliferation and label-retaining experiments

For label-retaining analyses, 10-day-old mice were injected with 50 mg kg^−1^ bodyweight EdU every 12 h for four injections. After a 10-day chase period, the mice were killed and dorsal skin was isolated for analysis. For short-term analysis of proliferation, 100 mg ml^−1^ BrdU (Sigma) was injected intraperitoneally 1 h before killing.

### Immunohistochemistry

Staining with haematoxylin/eosin was performed using standard protocols. For β-galactosidase staining skin biopsies were fixed in 0.2% glutaraldehyde in phosphate buffered saline (PBS) for 1 h at 4 °C. Tissue was washed with PBS and permeabilized with 0.2% NP-40 in PBS 1 h at room temperature, followed by incubation in staining solution (5 mM K-ferrocyanide, 5 mM K-ferricyanide, 2 mM MgCl_2_, 1 mg ml^−1^ X-Gal in 0.02% NP-40 in PBS) at 37 °C over night. Stained tissue was post-fixed with 4% paraformaldehyde and embedded in paraffin.

For alkaline phosphatase staining, frozen sections were fixed with acetone at -20 °C for 10 min, washed with PBS and stained with NBT/BCIP (Roche) in 0.1 M Tris-HCl, pH 9.5, 0.1 M NaCl. Sections were counterstained with Nuclear fast red (Roth) and mounted in Entellan (Merck).

pSmad2 (Cell Signaling 3101; 1:100) staining was detected using biotinylated secondary antibody (31820, Thermo Scientific; 1:500) followed by tertiary labelling with streptavidin-HRP (Dako, 1:300), staining with DAB chromogen substrate (Dako) and subsequent counterstain with haematoxylin.

Images were collected with a Leica DM4000 light microscope using a × 10 or × 20 objective.

### Immunofluorescence

Immunofluorescence was carried out as described previously^14^. Briefly, cells grown on glass coverslips or tissue pieces embedded in cryomatrix (Thermo Shandon) were fixed in 4% paraformaldehyde or ice-cold methanol, permeabilized with 0.3% Triton X-100 in PBS and blocked in 5% BSA. Stainings on paraffin sections was carried out after deparaffinization and antigen retrieval (20 min in a pressure cooker) in target retrieval solution (TRS; Dako).

Samples were subsequently incubated overnight in primary antibody in 1% BSA, followed by washing and incubation in secondary antibody (Alexa Fluor, Life Technologies; 1:500). Finally, samples were mounted in Elvanol. The following primary antibodies were used to stain cells and frozen sections: ILK (Cell Signaling; 1:500) LN-332 (gift from R.E. Burgeson; 1:20,000), LNα5 (ref. 62) (gift from L. Sorokin; 1:20,000), CD34 (gift from M. Koch; 1:1,000 or eBioscience clone RAM34; 1:250), P-Cadherin (Invitrogen; 1:200), K15 (Thermo Scientific; 1:2,000) and K14 (Covance; 1:1,000 or Progen; 1:100).

TRS pH 9 (TRS; Dako) was used for stainings on paraffin sections with antibodies against Sox9 (Santa Cruz, sc-20095; 1:100), BrdU (BD 347580; 1:25), Ki67 (Dako M7249, 1:100) and K14 (Covance; 1:1,000 or Progen; 1:100). TRS pH 6 (Dako) was used for staining with antibodies against Nfatc1 (Santa Cruz, sc-7294; 1:200), γH2AX (Cell Signaling, 1:250), β-catenin (Santa Cruz, sc-7199; 1:200), K14 (Covance; 1:1,000 or Progen; 1:100) and p53 (Leica CM5; 1:100).

For detection of EdU, paraffin sections were deparaffinized and incubated in 100 mM Tris pH 8.5, 1 mM CuSO_4_ and 100 mM ascorbic acid containing 10 μM 488-Azide (Invitrogen) for 30 min at room temperature. Apoptotic cells were detected by Terminal deoxynucleotidyl transferase dUTP nick end labeling (TUNEL) staining (Promega) according to the manufacturer's instructions.

Fluorescent images were collected by laser scanning confocal microscopy (TCS SP5X; Leica) with × 63 or × 40 immersion objectives using LAS software. All images were recorded sequentially and averaged at least twice. Fluorescent images were quantified using ImageJ software (NIH).

### FACS analysis

The epidermis was separated from the dermis using 0.8% trypsin (50 min at 37 °C), minced and filtered through 70-μm and 40-μm cell strainers. Single-cell suspensions were subsequently incubated with primary antibodies in 5% FCS in PBS for 45 min at 4 °C. FACS analysis was performed using a FACSCanto II cytometers equipped with FACSDiva Software (BD). The following antibodies were used: CD49f-FITC (BD 555735; 1:500), CD49f-eFluor450 (eBioscience; 1:300), CD34 (eBioscience clone RAM34; 1:100). Cell viability was assessed by 7AAD (eBioscience; 1:50). Data were analysed using FlowJo 7.6 software.

### RNA-seq of isolated bulge SCs

CD34+/α6 integrin^hi^ bulge SCs were isolated by FACS (FACSAria; BD) directly into TRIzol LS (Invitrogen), after which total RNA was isolated according to the manufacturer's instructions. Quality of the RNA for sequencing was determined using an Agilent 2200 TapeStation. Amplified complementary DNA (cDNA) was prepared at the Cologne Center for Genomics using Ovation RNA-Seq System V2 (NuGen) followed by subsequent library preparation using the Nextera XT library preparation kit (Illumina). RNA-sequencing was carried out on Illumina HiSeq2000 machines using the 2 × 100-bp protocol and V3 chemistry.

After quality control, adapter sequences were removed by flexbar^63^. Reads mapping to ribosomal RNA-related genes were filtered out using a custom ribosomal RNA-only reference. After preprocessing, reads were mapped to the Mus musculus reference genome (build GRCm38_79), followed by differential gene expression analysis using the DESeq2 R library (version 1.6.3). Transcripts regulated greater than twofold with an adjusted *P* value of ≤0.05 were used in GO term and KEGG pathway analysis (DAVID Bioinformatics Resources 6.7) to find enriched functional annotations.

### Magnetic cell separation

Single-cell suspensions of epidermis were generated as above. Cells were incubated with CD49f-FITC (BD 555735; 1:100) for 30 min at 4 °C. Cells were subsequently rinsed and incubated with anti-FITC antibodies coupled to magnetic beads (Miltenyi Biotech) for 20 min at 4 °C. CD49f(+) cells were separated and collected using a magnetic column (Miltenyi Biotech) and lysed for RNA isolation.

### Quantitative RT–PCR

Total RNA was isolated using the RNeasy Plus Mini Kit, after which cDNA was synthesized using the High-Capacity cDNA Reverse Transcription Kit (Applied Biosystems) or in case of FACS-sorted SCs using SuperScript Vilo (Invitrogen). qPCR was performed on the StepOne Plus (Applied Biosystems) or CFX384 (Bio-Rad) Real-Time PCR systems using the DyNAmo ColorFlash SYBR Green Mix (Thermo Fisher). Gene expression changes were calculated following normalization to GAPDH, S26, S18 or β-actin using the comparative Ct (cycle threshold) method. For a complete list of primers used, see Supplementary Table 1.

### Isolation of primary keratinocytes and adhesion signalling experiments

Single-cell suspensions of P21 epidermis were generated as above. Keratinocytes were plated and cultured feeder-free in keratinocyte growth medium containing 8% calcium-depleted FCS and low calcium (45 μM) and directly used for experiments.

For adhesion on defined ECM substrates, LN-511 (10 μg ml^−1^), LN-332 (1  μg ml^−1^; both from Biolamina) and collagen I (25  μg ml^−1^)+fibronectin (10 μg ml^−1^; both from Millipore) were coated on tissue culture dishes overnight at 4 °C. Unspecific binding was blocked with heat-inactivated 2% BSA in PBS for 1 h at room temperature, after which freshly isolated cells were allowed to adhere 6 h before harvesting.

For generating a cell-free ECM platform, primary wild-type human keratinocytes (CellNTec) were cultured in serum-free medium (CellNTec) on tissue culture dishes or glass coverslips for 6 days. Cells were removed by incubating the cultures in extraction buffer (20 mM ammonium hydroxide and 0.5% Triton X-100 in PBS) at 37 °C. The cell-free matrix was subsequently rinsed with PBS and blocked with heat-inactivated 2% BSA in PBS for 1 h at room temperature. Freshly isolated keratinocytes were plated and allowed to adhere on the matrices for 12 h before harvesting. For siRNA-mediated depletion of LAMA3 and LAMA5, keratinocytes were transfected with Silencer Select siRNAs (s8060 and s8061 for LAMA3, s8065 and s8066 for LAMA5; AM4635 for scrambled; Ambion) using Lipofectamine RNAiMax transfection reagent (Invitrogen) according to the manufacturer's instructions. At 72 h after transfection, the keratinocytes were detached using accutase (Sigma) and replated, after which the transfection procedure was repeated. At 72 h after the second transfection, cell-free ECM platforms were prepared as above.

### Preparation of cell and tissue extracts and western blot

For epidermal tissue extracts, the epidermis was separated from the dermis by floating skin biopsies in 0.5 M ammonium thiocyanate (NH_4_SCN) in phosphate buffer (0.1 M Na_2_HPO_4_ and 0.1 M KH_2_PO_4_, pH 6.8) for 40 min on ice.

Epidermal extracts were suspended in lysis buffer (50 mM Tris-HCl buffer (pH 8.0), 150 mM NaCl, 1% Triton X-100, 0.05% sodium deoxycholate, 10 mM EDTA, protease and phosphatase inhibitors). Cultured cells were harvested directly in lysis buffer.

For ECM extraction from whole skin, tissue biopsies were pulverized using a mortar and pestle in liquid nitrogen. All subsequent steps were performed at 4 °C with solutions containing protease and phosphatase inhibitors. Ground tissues were suspended in Tris-buffered saline (50 mM Tris-HCl, 150 mM NaCl, pH 7.4). The samples were washed in the same buffer to remove serum proteins. ECM proteins were first solubilized by stirring the tissues in 20 mM EDTA in Tris-buffered saline for 2 h, followed by overnight incubation in 4 M urea. Next, the samples were centrifuged and the supernatant was collected and analysed.

All samples were reduced in Laemmli sample buffer at 95 °C, separated by SDS–polyacrylamide gel electrophoresis, transferred onto polyvinylidene difluoride or nitrocellulose membranes and subjected to western blot analysis using standard protocols. The following primary antibodies were used: β-catenin (Santa Cruz, sc-7199; 1:5,000), pSmad2 (Cell Signaling, 3101; 1:1,000), Smad2 (Cell Signaling, 3103; 1:1,000), pSmad 1/5/8 (Cell Signaling, 9511; 1:1,000), Ras (BD 610001; 1:2,500), ILK (BD 611802; 1:2,500), LNγ2 (Santa Cruz, sc-7652; 1:500), LNγ1 (Santa Cruz, sc-5584; 1:500), Actin (Millipore, MAB1501; 1:100,000) and GAPDH (Calbiochem, 1001; 1:100,000). For quantification, all samples were loaded in duplicate and fractionated at least twice on independent gels. Band intensity from X-ray films was measured using ImageJ software.

### Two-stage skin carcinogenesis experiment

Cutaneous two-stage chemical carcinogenesis experiments were performed as previously described^37^. Eight-week-old mice were treated twice with topical application of 100 nmol 7,12-dimethylbenz[a]anthracene (DMBA; Sigma) in 100 μl of acetone. At 2 weeks post DMBA, 10 nmol 12-*O*-tetradecanoylphorbol-13-acetate (TPA; Sigma) in 200 μl of acetone was applied biweekly for 18 weeks. The experiment was terminated after 18 weeks of TPA due to a skin health condition of ILK-K5 mice. Tumour size and number were recorded weekly after the start of TPA treatment (week 0). All mice were euthanized at the end of the experiment. Skin lesions were analysed by histology.

### Statistical analyses

Statistical analyses were performed using GraphPad Prism software (GraphPad, version 5.0). Statistical significance was determined by the Mann–Whitney *U*-test, Wilcoxon matched-pairs signed-rank test, analysis of variance with Bonferroni's *post hoc* test, Student's *t*-test or *χ*^2^-test. The test used for each experiment is indicated in the corresponding figure legend. When a test for normally distributed data was used, normal distribution was confirmed with the Kolmogorov–Smirnov test (*α*=0.05).

## Additional information

**Accession codes:** RNA-seq data have been submitted to NCBI-GEO (GSE68846).

**How to cite this article:** Morgner, J. *et al.* Integrin-linked kinase regulates the niche of quiescent epidermal stem cells. *Nat. Commun.* 6:8198 doi: 10.1038/ncomms9198 (2015).

## Supplementary Material

Supplementary Information Supplementary Figures 1-7 and Supplementary Table 1

Supplementary Data 1 List of genes regulated in ILK-K5 HFSCs
