# Supplementary material for: Integrin-linked kinase regulates the niche of quiescent epidermal stem cells
Source: Nat Commun. 2015 Sep 8;6:8198. doi: 10.1038/ncomms9198 (PMC4569844; doi:10.1038/ncomms9198)
Supplement: Supplementary Information — Supplementary Figures 1-7 and Supplementary Table 1 [file ncomms9198-s1.pdf]

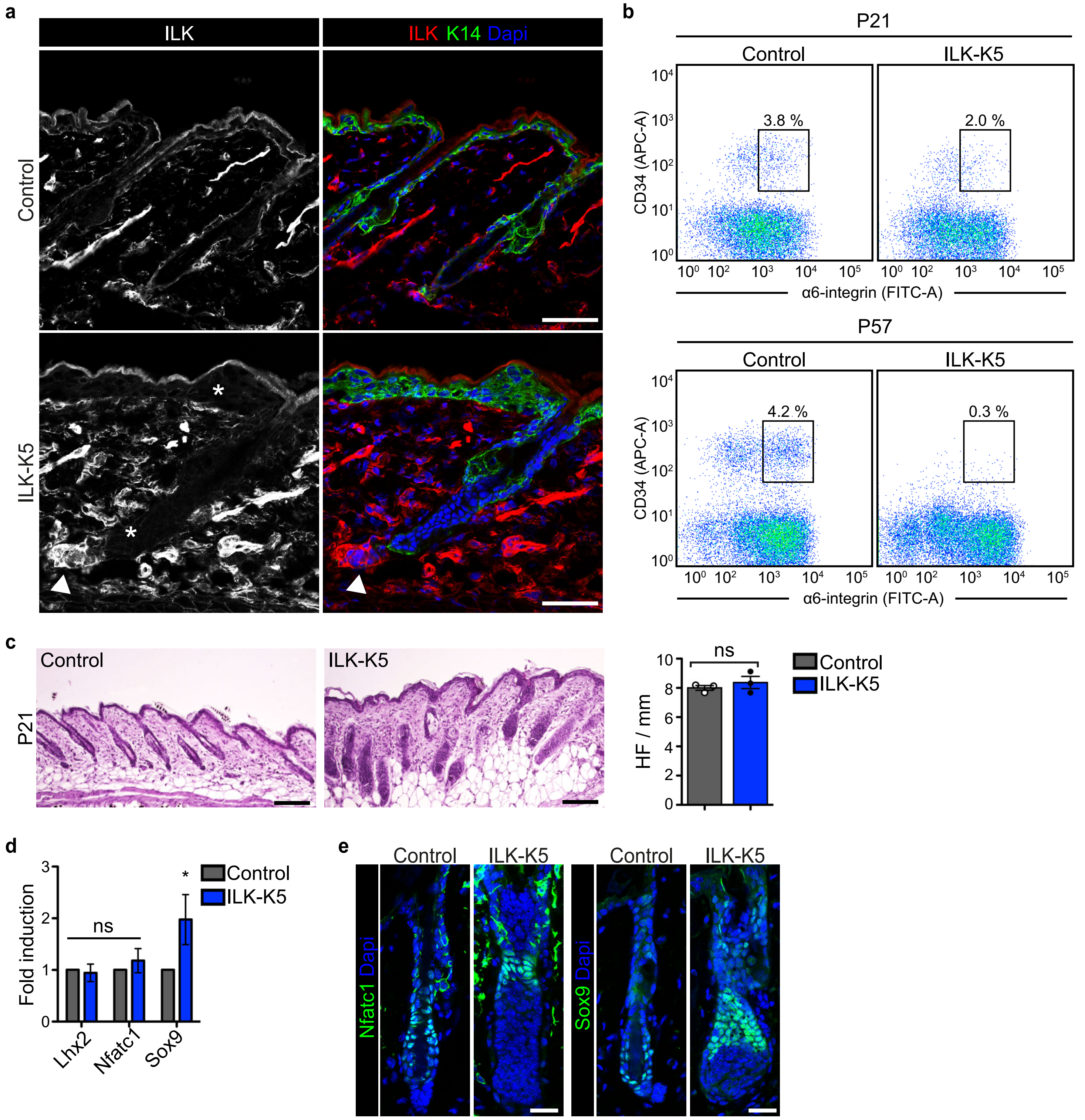

Supplementary Figure 1. Analysis of stem cell (SC) number, hair follicle density and SC fate-determining factors in ILK-K5 mice

(a) Immunofluorescence staining for ILK (red) and K14 (green). ILK staining is not detected in hair follicles (HFs) and interfollicular epidermis (IFE) (asterisks) of ILK-K5 mice at P21, whereas the dermal papilla (DP) is positive for ILK (arrowhead). Scale bar 50 μm.

(b) Representative plots of FACS analyses with antibodies against CD34 and α6 integrin from P21 and P57 epidermis. ILK-K5 mice show reduced amounts of $CD34^{+}/\alpha6^{hi}$ HFSCs.

(c) Hematoxylin/eosin staining of P21 skin (scale bars 100 μm). No difference in HF density between control and ILK-K5 mice is observed (mean ± SEM; n=3; ns=not significant, p=0.70, Mann-Whitney).

(d) RT-qPCR analysis of key transcription factors required to establish bulge SC fate. No significant difference is observed in expression of Lhx2 or Nfatc1. Sox9 expression is slightly increased (mean ± SEM; n=4; ns=not significant, p>0.2817; *p=0.0211, Mann-Whitney).

(e) Immunofluorescence staining for Nfatc1 (left) and Sox9 (right) at P21. Scale bar 25 μm.

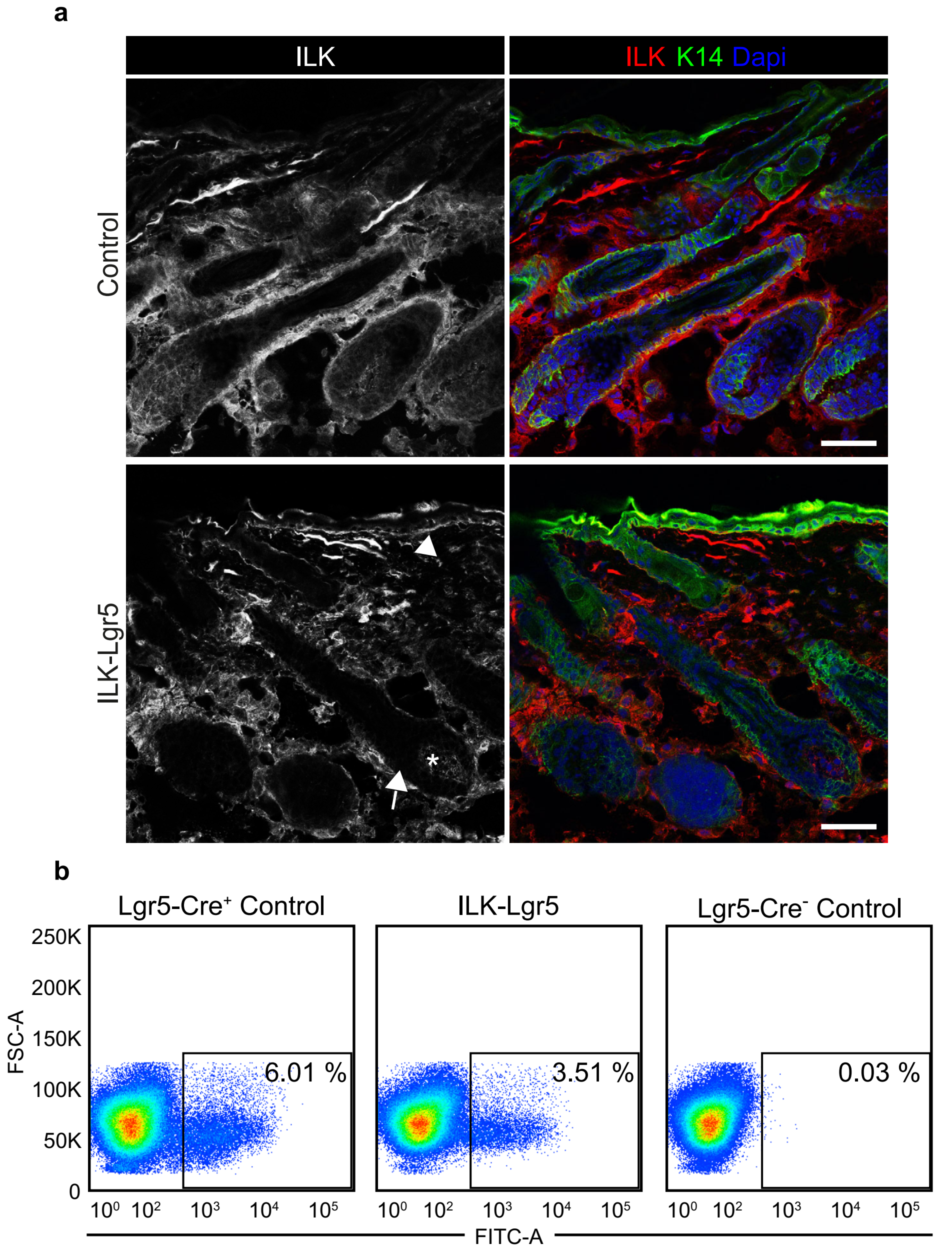

Supplementary Figure 2. Analysis of ILK expression and Lgr5-EGFP cells in ILK-Lgr5 mice
(a) Immunofluorescence staining for ILK (red) and K14 (green). ILK staining is not detected in HFs of ILK-Lgr5 mice (arrows), whereas the IFE (arrowhead) and DP (asterisk) show comparable staining to control mice. Scale bars 50 µm.
(b) Representative FACS plots for quantification of Lgr5-EGFP cells. ILK-Lgr5 mice show reduced amounts of EGFP-positive cells.

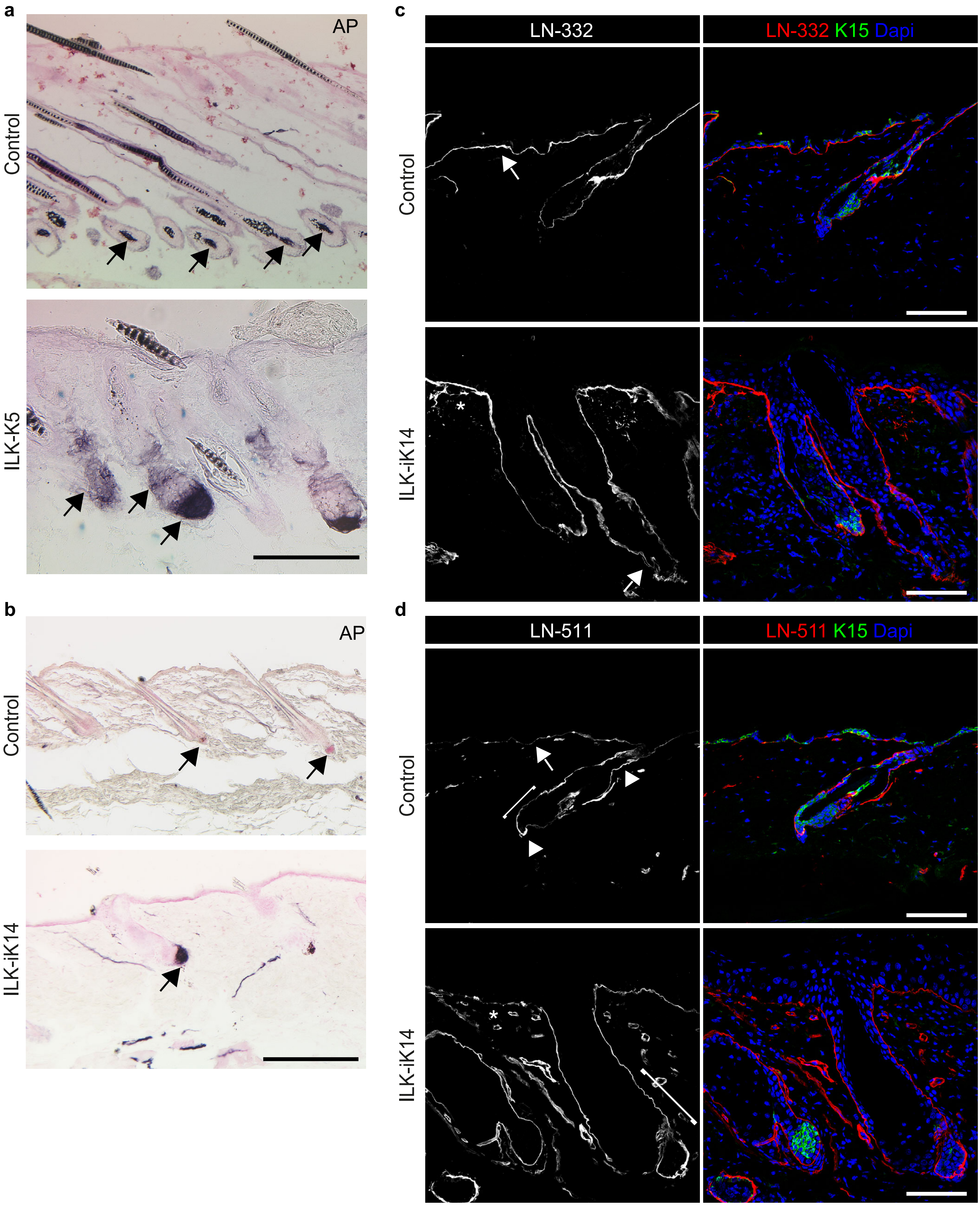

Supplementary Figure 3. Analysis of DP and BM in ILK-K5 and ILK-iK14 mice
(a) Alkaline phosphatase (AP) staining to detect DP cells in ILK-K5 skin during anagen (P14). In control skin the DP is encapsulated by the HFs (arrows; upper panel), whereas in ILK-K5 skin AP-positive cell population is increased and surrounds the base of the HF (arrows; lower panel). Scale bars 200 µm. **(b)** AP staining to detect DP cells in ILK-iK14 skin after 8 months of doxycycline. In control skin the DP is attached to the base of telogen HFs (arrows; upper panel), whereas in ILK-iK14 skin the AP-positive cell population is increased and encapsulates the base of the HF (arrows; lower panel). Scale bars 200 µm. **(c)** Immunofluorescence staining for LN-332 (red) and K15 (green). LN-332 staining shows higher intensity beneaththe IFE (arrow) than around HFs in control skin (upper panel). Note fragmentation of LN-332-stained BM in the IFE (asterisk) and tips of HFs (arrow) in ILK-iK14 skin (lower panel). Scale bars 50 µm. **(d)** Immunofluorescence staining for LN-511 (red) and K15 (green). LN-511 staining shows highest intensity at the isthmus region and around HG (arrowheads). Only faintstaining is observed beneath the IFE (arrow) and around bulge (bracket) in control skin (upper panel). Note fragmentation ofLN-511-stained BM in the IFE (asterisk) and high intensity around bulge and HG (brackets) in ILK-iK14 skin (lower panel). Scale bars 50 µm.

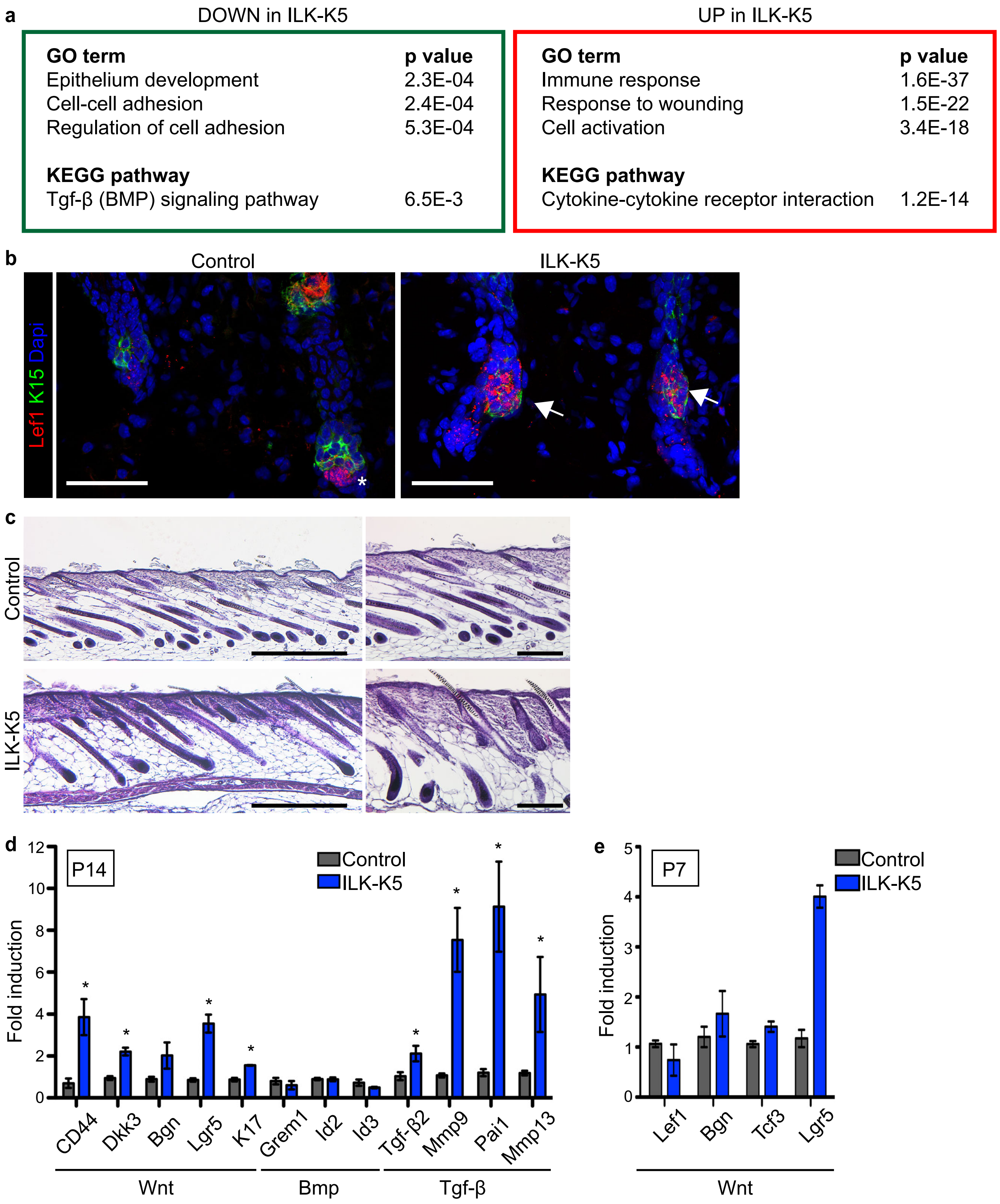

Supplementary Figure 4. Analysis of HFSC activation status in ILK-K5 mice
(a) Summary of GO term and KEGG pathway analyses from RNA-seq data of FACS sorted bulge SCs.
(b) Lef1 staining (red) from P21 control and ILK-K5 skin. Controls show staining in the DP only (asterisk), whereas in ILK-K5 mice also K15-positive cells (green) show staining for Lef1. Scale bar 50 µm.
(c) Hematoxylin/eosin staining of P14 skin. Both control and ILK-K5 HFs display anagen morphology. Scale bars 500 µm/left panel; 100 µm/right panel.
(d) RT-qPCR analysis shows upregulation of Wnt and Tgf-β pathway target genes, but no change in Bmp pathway target genes in sorted EPCs from P14 ILK-K5 skin (mean ± SEM; n=3, *p<0.05, Mann-Whitney).
(e) RT-qPCR analysis of Wnt target gene expression from P7 EPCs (mean ± SEM; n=4; p>0.0765, Mann-Whitney).

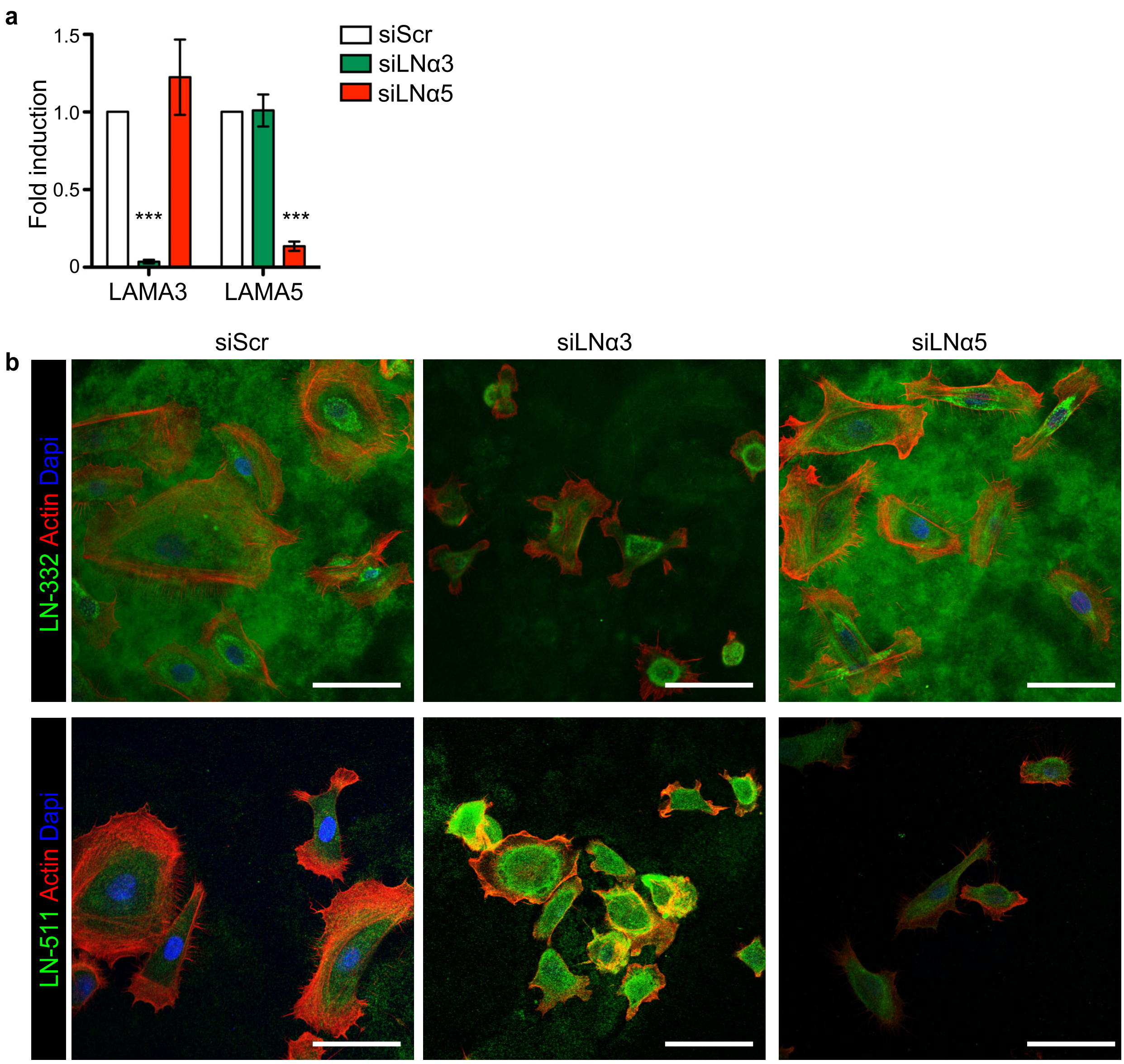

Supplementary Figure 5. Analysis of LN expression and deposition in keratinocytes transfected with LNα3 or α5 siRNA

(a) RT-qPCR analysis of LNα3 and LNα5 in cells transfected with scrambled (siScr), LNα3, or LNα5 siRNA. Note efficient and specific depletion of the respective LNα-chain upon siRNA transfection (mean ±SEM; n=4, ***p<0.001, 2-Way ANOVA).

(b) Immunofluorescence staining for actin (red) and LN-332 or LN-511 (both in green). Depletion of LNα3 leads to reduced deposition of LN-332, whereas depletion of LNα5 has no effect on LN-332 deposition (upper panel). Cells transfected with siScr deposit very little LN-511 (lower panel). Depletion of LNα5 leads to reduced deposition of LN-511. In addition, depletion of LNα3 leads to a sligh increase in LN-511 deposition (lower panel). Scale bars 50 µm.

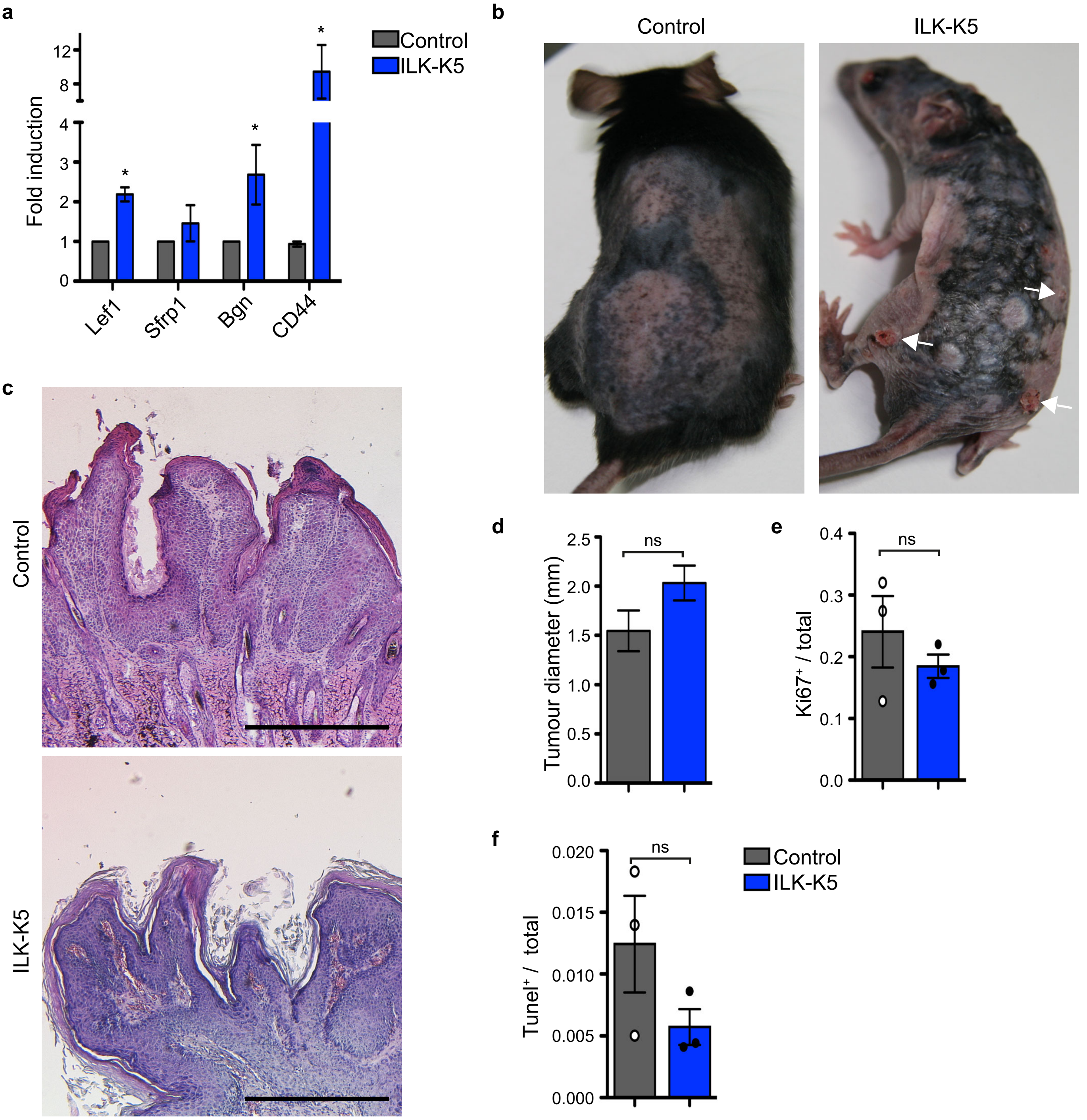

Supplementary Figure 6. Analysis of Wnt target gene expression and tumour development in ILK-K5 mice
(a) RT-qPCR analysis shows upregulation of Wnt target gene expression in sorted EPCs from P57 ILK-K5 skin (mean ± SEM; n=4; *p=0.0211, Mann-Whitney).
(b) Representative images of mice treated twice with DMBA followed by 18 weeks of TPA. All ILK-K5 mice had developed papillomas (arrows) at this point.
(c) Hematoxylin/eosin stainings of papillomas from control and ILK-K5 mice show comparable histology. Scale bars 500 µm.
(d) Quantification of tumour diameter shows no significant difference in tumour sizes from control and ILK-K5 mice (mean ± SEM; n=11/31; ns=not significant, p=0.1386, Student's t-test).
(e) Quantification of proliferation within tumours using the marker Ki67 shows no significant difference in proliferation between control and ILK-K5 tumours (mean ±SEM; n=3; ns=not significant, p=0.70, Mann-Whitney).
(f) Quantification of apoptosis within tumours using Tunel staining shows no significant difference in tumour cell apoptosis in control and ILK-K5 mice (mean ± SEM; n=3; p=0.344, Mann-Whitney).

Supplementary Figure 7. Full scans of Western blots in this study

Fig. 4c

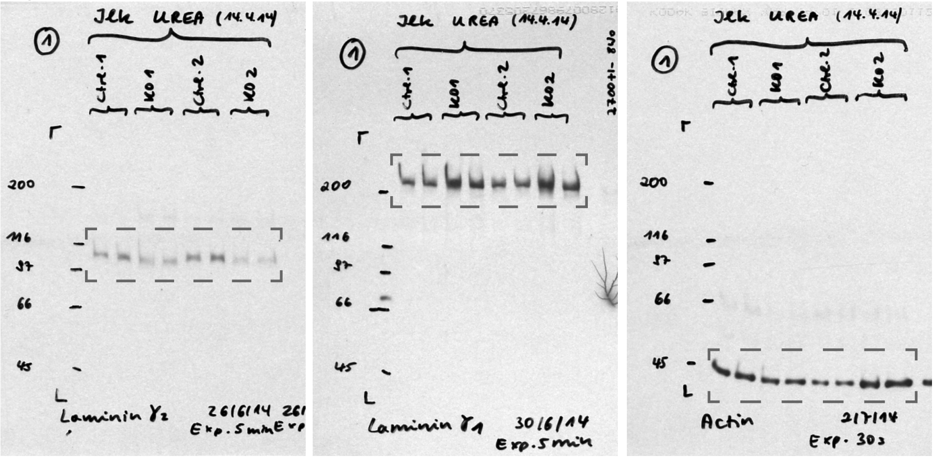

Fig. 5f

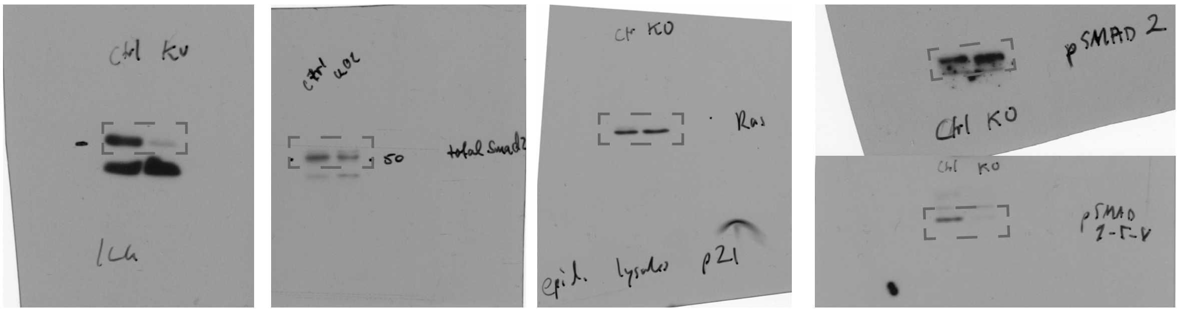

Fig. 6a

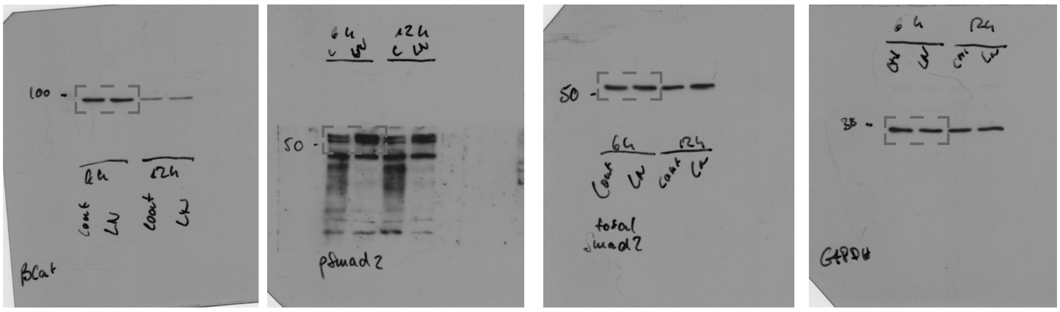

Fig. 6c

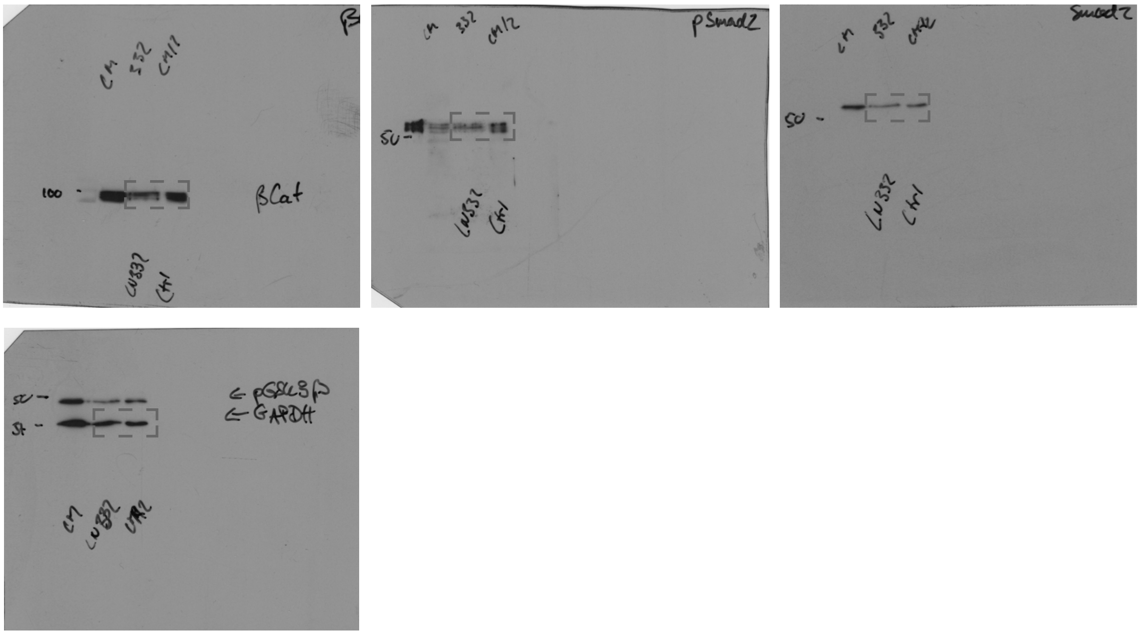

Supplementary Table 1 Primer sequences used for qRT-PCR

**Gene/oligo**	**Sequence 5’ – 3’**	**Amplicon lenght (bp)**
mβAct fw	TCAAGATCATTGCTC	106
mβAct rev	TACTTCTGCTTGCTGATCCAC	
mAxin2 fw	AGCGCCAACGACAGCGAGTT	188
mAxin2 rev	AGGCGGTGGGTTCTCGGAAA	
mBambi fw	CTTTGGAATGCTGTCACGAA	149
mBambi rev	GGAAGTCAGCTCCTGCATCT	
mBgn fw	TCCGCACTCCAACAACATCA	204
mBgn rev	GGCAACCACTGCCTCTACTT	
mCD44 fw	AGCCCCTCCTGAAGAAGACT	116
mCD44 rev	ACTCGCCCTTCTTGCTGTAG	
mDkk3 fw	ATGCTATGCACCCGAGACAG	159
mDkk3 rev	GAACAGCAGGCCTCTTTGGA	
mGAPDH fw	GGTGTGAACGGATTTGGCCGTATTG	155
mGAPDH rev	CCGTTGAATTTGCCGTGAGTGGAGT	
mGrem1 fw	CCACGGAAGTGACAGAATGA	141
mGrem1 rev	TTGTGCTGAGCCTTGTCAGG	
mId2 fw	ATCCCCCAGAACAAGAAGGT	128
mId2 rev	TGTCCAGGTCTCTGGTGATG	
mId3 fw	GCATGGATGAGCTTCGATCT	126
mId3 rev	ACCAGCGTGTGCTAGCTCTT	
mK17 fw	GAGAGGATGCCCACCTGACT	138
mK17 rev	GTCCTTAACGGGTGGTCTGG	
hLAMA3 fw	AGTTCACAGCAGCAAAGGGT	211
hLAMA3 rev	GCTGACAGTTAACACATATGCCTG	
hLAMA5 fw	CTTCGTCTTCTACGTCGGGG	109
hLAMA5 rev	CACCTCCTCATTCAGCGTGT	
mLef1 fw	CGGAACTCTGCGCCACCGAT	177
mLef1 rev	TGACCACCTCATGCCCGTTGC	
mLhx2 fw	CCTACTACAACGGCGTGGGCACTGT	137
mLhx2 rev	GTCACGATCCAGGTGTTCAGCATCG	
mLgr5 fw	CCAATGGAATAAAGACGACGGCAACA	128
mLgr5 rev	GGGCCTTCAGGTCTTCCTCAAAGTCA	
mMmp9 fw	TGAGCTGGACAGCCAGACACTAAA	148
mMmp9 rev	TCGCGGCAAGTCTTCAGAGTAGTT	
mMmp13 fw	TGTTTGCAGAGCACTACTTGAA	132
mMmp13 rev	CAGTCACCTCTAAGCCAAAGAAA	
mNfatc1 fw	GGTGCTGTCTGGCCATAACT	128
mNfatc1 rev	CCAGGGAATTTGGCTTGCAC	
mPai1 fw	GACACCCTCAGCATGTTCATC	218
mPai1 rev	AGGGTTGCACTAAACATGTCAG	
mS18 fw	GATCCCAGACTGGTTCCTGA	79
mS18 rev	GTCTAGACCGTTGGCCAGAA	
hS26 fw	GCGAGCGTCTTCGATGCCTATGT	128
hS26 rev	GGGGGTGTTCGGTCCTTGCG	

mS26 fw	CGTCTTCGACGCCTACGTGCT	180
mS26 rev	CGGCCTCTTTACATGGGCTTTGGT	
mSfrp1 fw	GCAAGCGAGTTTGCACTGAGGATGA	101
mSfrp1 rev	GGCCCCAGCTTCAAGGGTTTCTTCT	
mTcf3 fw	CTCAGCAGCAAATCCAAGAGGCAGAG	109
mTcf3 rev	TGGGAAGACGCAGGGCTATCACAAG	
mTgfb2 fw	GAACCCAAAGGGTACAATGC	100
mTgfb2 rev	TGGTGTTGTACAGGCTGAGG	
